# Structural brain imaging in Alzheimer’s disease and mild cognitive impairment: biomarker analysis and shared morphometry database

**DOI:** 10.1038/s41598-018-29295-9

**Published:** 2018-07-26

**Authors:** Christian Ledig, Andreas Schuh, Ricardo Guerrero, Rolf A. Heckemann, Daniel Rueckert

**Affiliations:** 1 Imperial College London, Department of Computing, London, SW7 2AZ UK; 2 MedTech West, Sahlgrenska University Hospital, 413 45 Gothenburg, Sweden; 3 Department of Radiation Therapy, Sahlgrenska Academy, University of Gothenburg, Gothenburg, Sweden; 4 Division of Brain Sciences, Imperial College London, London, UK

## Abstract

Magnetic resonance (MR) imaging is a powerful technique for non-invasive *in*-*vivo* imaging of the human brain. We employed a recently validated method for robust cross-sectional and longitudinal segmentation of MR brain images from the Alzheimer’s Disease Neuroimaging Initiative (ADNI) cohort. Specifically, we segmented 5074 MR brain images into 138 anatomical regions and extracted time-point specific structural volumes and volume change during follow-up intervals of 12 or 24 months. We assessed the extracted biomarkers by determining their power to predict diagnostic classification and by comparing atrophy rates to published meta-studies. The approach enables comprehensive analysis of structural changes within the whole brain. The discriminative power of individual biomarkers (volumes/atrophy rates) is on par with results published by other groups. We publish all quality-checked brain masks, structural segmentations, and extracted biomarkers along with this article. We further share the methodology for brain extraction (pincram) and segmentation (MALPEM, MALPEM4D) as open source projects with the community. The identified biomarkers hold great potential for deeper analysis, and the validated methodology can readily be applied to other imaging cohorts.

## Introduction

Non-invasive magnetic resonance (MR) brain imaging can support the quantitative characterization of neurological conditions such as Alzheimer’s disease (AD). MR imaging can provide informative biomarkers even before clinical symptoms are apparent or irreversible neuronal damage has occurred^1,2^. The diagnostic potential of biomarkers based on structural imaging has been outlined by Frisoni *et al*.^3^ and Klöppel *et al*.^4^. Automatically extracted biomarkers can provide diagnostic decision support, increase objectivity in the disease assessment and improve differential diagnosis^3–6^. Another important avenue is the use of biomarkers for AD screening or for enrolling suitable participants for pharmaceutical trials^4,7^. Clinical trials can also benefit from MR biomarkers as they enable enrichment strategies^8^ or more rigorous inclusion criteria, leading to more homogeneous study groups^1–4^.

**Figure 1:**
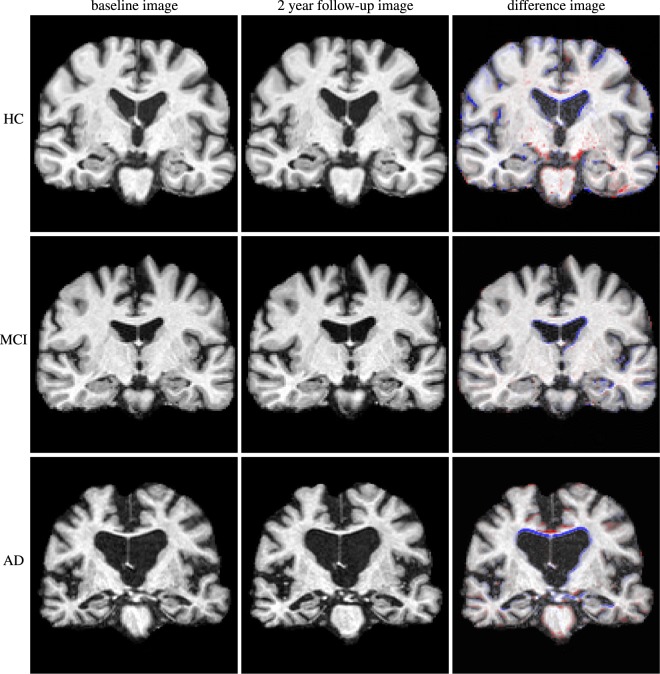
Three examples of MR images (brain-extracted) of subjects from the ADNI1 cohort in coronal section. Top row: a healthy control subject (male, 84.8 years at baseline); middle row: MCI subject (female, 71.8 year at baseline) who converted to AD after three years; bottom row: an AD patient (male, 77.5 years at baseline). Left: baseline; middle: 2-year follow-up; right: baseline with overlaid difference image of rigidly aligned images (blue: volume loss/atrophy, red: positive volume change). The differences are visually subtle, but the increased atrophy in the medial temporal lobe and the enlarged ventricles are apparent in the difference image.

Subjects with mild cognitive impairment (MCI) do not fulfil the diagnostic criteria for AD^9^, but are at increased risk of developing AD^10^. Predicting conversion to AD is of particular importance to patients, clinicians and caregivers, but also for clinical trials^4,9,10^. An illustration of anatomical changes over a period of two years is shown in Fig. 1 for a healthy control (HC) subject, a progressive MCI (pMCI) subject converting to AD and a patient with AD. Modern neuroimaging can help to improve the accuracy of MCI diagnosis by adding positive predictive value when combined with other diagnostic criteria^5,9,11,12^. It is, however, uncertain whether information based on *individual* brain structures is sufficient to fully characterize the complex progression of AD or even to enable a differential dementia diagnosis^7,13^. Recent studies further suggest that structural MR imaging in combination with other diagnostic procedures, such as positron emission tomography (PET) or chemical analysis of cerebrospinal fluid (CSF), can detect pathological AD-related change years before the onset of AD dementia^3,14^. Many studies have shown that with progression of the disease, there is significant atrophy in structures of the medial temporal lobe (MTL) such as the hippocampus, amygdala, and entorhinal and parahippocampal cortices^4,15–17^. In the future, structural MR imaging will thus play an important role not only in the diagnosis of AD, but also in monitoring its treatment^2–4^. The development of automatic, robust, quantitative techniques to assess MR images of the brain is therefore an important factor to further increase the utility of structural imaging in the context of neurocognitive disorders.

A vast number of studies have shown correlations between quantitative measures calculated from brain MR images with AD progression. Automatic methods perform similarly to trained radiologists when classifying MR images of patients with AD^5^. There is strong evidence that different anatomical brain structures are affected at different stages of the disease^18^, with early involvement of the hippocampus, amygdala and entorhinal cortex consistently shown in the literature^9,15,17^. Although sensitive to dementia, these markers may yet not be sufficiently specific to AD^3,12^. A uniform approach that considers pathological changes of multiple structures within the *whole* brain promises to increase specificity in dementia diagnosis and to support differential assessment of various types of dementia^3,13^. It is thus desirable to follow a holistic approach and to analyse a large number of structures of the whole brain rather than only a limited selection of brain structures. Due to its early involvement in dementia, the focus of many published methods lies on the segmentation of the hippocampus to quantify its volume or shape^11,18–29^. Grey matter (GM) tissue maps^10,13^ and cortical thickness have also been shown to be of high predictive value in the context of AD^12,30^. Other approaches are based on voxel-based morphometry (VBM)^31^, deformation-based morphometry (DBM)^32^, or tensor-based morphometry (TBM)^33^ to study group differences. In general, methods exploring the whole brain outperform those focusing on individual structures such as the hippocampus^12,34^.

Many studies have shown that biomarkers of morphometry, such as volume or shape, correlate with AD progression. However, intersubject variability can lead to substantial overlap with the healthy population and thus limit the discriminative power of these features^21^. *Temporal change* of the whole brain or individual structures tends to be more consistent between subjects. Atrophy rates are usually given as a percentage per year. Popular approaches rely on 3D + t optimization using graph cuts^22^, expectation maximization^35^, or the boundary shift integral^21,23,36^. The measurable increase in GM atrophy in patients with AD is a consequence of a substantially accelerated, regionally selective loss of neurons^20,37^. Most of the published studies can, however, only be compared qualitatively. In many cases, different features, a different methodology to extract the features and different classification techniques are used^7^. In addition, methods are applied to different cohorts or different subsets thereof. Most studies lack histopathologically confirmed ground truth diagnoses. Instead of seeing subjects as being either healthy or diseased, the diagnosis of AD is a dynamic process in which biomarkers gradually begin to change before current diagnosis criteria are met^38–44^. Advances in machine learning, e.g. Gaussian process modelling, have also introduced novel opportunities for personalized healthcare, shifting from “one-size-fits-all” population modeling towards personalized models^45–48^.

Of particular importance for routine use in clinical practice is the interpretability of biomarkers^7^. Many recently developed methods rely heavily on machine learning techniques, e.g. learned manifolds^34,49^, multiple instance learning^50^, or region grading^51^. Even though these methods are often highly accurate, the interpretation of their results can be difficult, and this impedes their adoption into clinical practice. Thus, it is desirable to calculate biomarkers that are easy to interpret, but at the same time as informative as features obtained through such sophisticated machine learning techniques. A further overview can be found in numerous surveys^1,3,7,12,52^.

In this manuscript we employ multi-atlas label propagation with expectation-maximisation based refinement (MALPEM)^53^, a state-of-the-art automatic segmentation method for robust segmentation of whole-brain MR images into 138 distinct anatomical structures. Johnson *et al*.^54^ recently validated a number of established segmentation methods (SPM^31,55^, ANTs Atropos^56^, MALP-EM^53,57^, FSL FAST^58^, FreeSurfer^59^) in the context of Huntington’s disease and found that “MALP-EM appeared to be the most visually accurate tool, […]”. In 2015, an entry based on MALPEM won a third prize in the CADDementia disease classification challenge held in conjunction with MICCAI^52^. We applied MALPEM to a set of 5074 images of the Alzheimer’s Disease Neuroimaging Initiative (ADNI) cohort with the goal to identify biomarkers that characterize the whole brain, specifically structural volumes and atrophy rates. Our main contributions are:

- We confirm the accuracy and robustness of MALPEM/MALPEM4D in a cross-sectional/longitudinal study based on a large number of images from the ADNI database.
- We assess the quality of extracted biomarkers with a clear clinical interpretation (volumes/atrophy rates) and show that their discriminative value is on par with published literature in the context of AD.
- We share the employed methodology for brain extraction (pincram) and segmentation (MALPEM, MALPEM4D) as open source projects. The validated methodology can readily be applied to other imaging cohorts.
- We share quality-checked brain masks, structural segmentations and extracted biomarkers for 5074 ADNI images with the community. This resource holds great potential for a deeper analysis and enables training of sophisticated model-based approaches by interested research groups.

## Results

### Cross-sectional analysis

**Figure 2:**
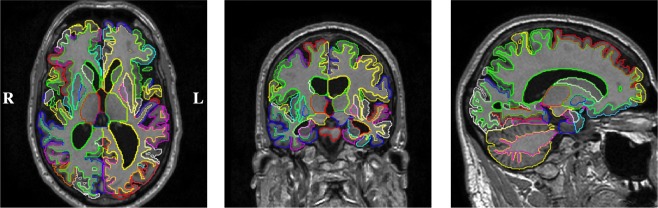
Example cross-sectional segmentation results of a patient diagnosed with AD (ADNI_018_S_0286, male, 66 years of age) in axial (left), coronal (middle) and sagittal (right) view-plane.

**Figure 3:**
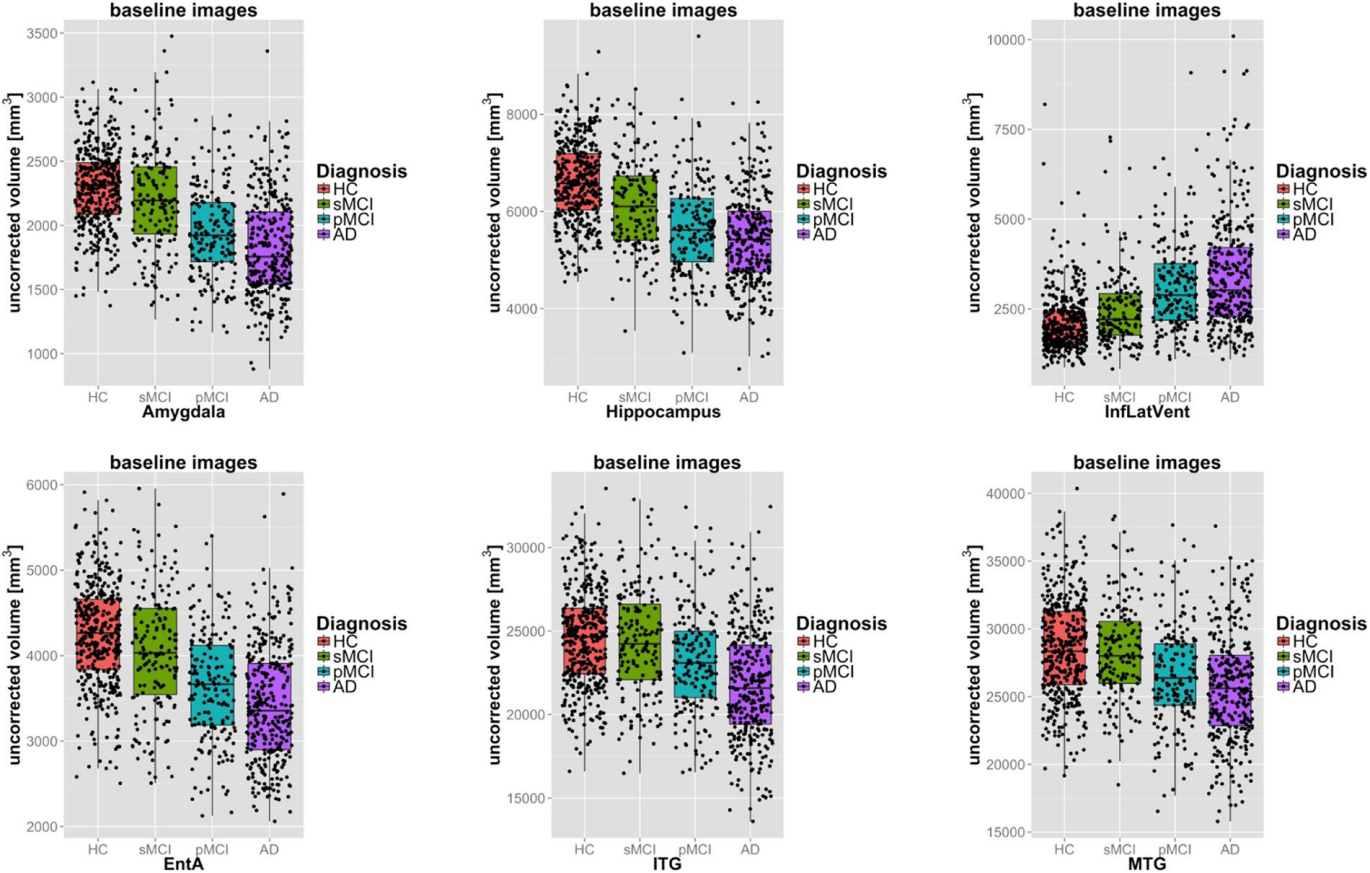
Boxplots of structural volumes at baseline for six selected structures before correcting for nuisance factors for distinct disease groups. Structures were selected based on their performance in classifying the investigated disease groups (c.f. Table 1).

In a cross-sectional analysis, we investigated the potential to discriminate AD disease stages based on the volumes of individual brain regions. An example segmentation result of a healthy control subject and a patient diagnosed with AD is shown in Fig. 2. The distribution of the measured s of six selected structures is shown in Fig. 3 for the four disease groups: HC, stable MCI (sMCI), pMCI and AD. The reduced GM volume of structures in the medial temporal lobe and the increased ventricular volume in patients with AD is apparent. All cross-sectional volumetric measurements were corrected for the nuisance variables subject age, gender, and intracranial. In the following, these corrected volumes will be employed to investigate their potential to classify relevant disease stages in AD.

#### Disease classification using structural volumes

An overview of the results of the classification experiments is given in Table 1. The volume of the amygdala allows the strongest distinction between AD and HC with an effect size (Cohen’s d) of 1.56 and a balanced classification accuracy (bACC) of 80%, a sensitivity (SENS) of 76% and a specificity (SPEC) of 84%. Other structures in the MTL region such as the hippocampus (bACC: 78%) or the entorhinal area (bACC: 78%) were similarly discriminative (d > 1.5). Established findings that total brain volume, and cortical grey matter in particular, are smaller in patients with AD were confirmed in our experiments. Accuracy was further improved by combining all structural volumes as features in a support vector machine (SVM; bACC: 89%, SENS: 86%, SPEC: 92%) or random forest (RF; (bACC: 86%, SENS: 83%, SPEC: 90%). As indicated in Table 1, numerous structural differences were highly significant even after Bonferroni correction for multiple comparisons. The structural volumes of the amygdala (bACC: 65%, SENS: 63%, SPEC: 68%) and inferior lateral ventricle (bACC: 64%, SENS: 54%, SPEC: 74%) were most discriminative for the classification of pMCI vs. sMCI. Combining all extracted structural volumes into a single RF classifier further increased classification accuracies (bACC: 68%, SENS: 72%, SPEC: 64%). The classification results for all structures can be found in the supplementary material.

**Table 1:** Classification results in % (6-fold cross-validation, LDA 100 runs, RF/SVM 20 runs) obtained distinguishing between AD and HC (top) and sMCI from pMCI (bottom).

AD patients (N = 322, Positives^P^) vs. Healthy Controls (N = 404, Negatives^N^) (baseline analysis, ^†^volumes corrected for age/gender/brain size)								
structure	ACC (bACC)	SENS	SPEC	mean [rel. to HC] (SD) [mm^3^]^P,†^	mean (SD) [mm^3^]^N,†^	effect size (d)	p-value	sig. (corr.)
RandomForest (all features)	87 (86)	83	90					
SVM (all features)	90 (89)	86	92					
(surrogate structures)								
BrainTissue	72 (71)	63	78	−17942.8 [$\hat{=}$ −1.5%] (22718.7)	0 (17403.6)	0.900	<0.00001	++ (++)
CorticalGreyMatter	68 (67)	63	72	−23635.9 [$\hat{=}$ −4.6%] (28907.3)	0 (24078.1)	0.898	<0.00001	++ (++)
Ventricles	72 (71)	63	79	17757.3 [$\hat{=}$ 46.2%] (22639.6)	0 (17293.9)	0.895	<0.00001	++ (++)
WhiteMatter	57 (56)	51	61	7242.2 [$\hat{=}$ 1.7%] (29309.8)	0 (28686.1)	0.250	0.00086	++ (o)
DeepGreyMatter	52 (52)	50	54	−1549.1 [$\hat{=}$ −0.9%] (13310.2)	0 (11142.2)	0.127	0.08834	o (o)
Brain	54 (55)	56	53	1199158.5 (132290.9)	1212115.6 (118623.9)	0.104	0.16526	o (o)
(selected individual structures)								
Amygdala	80 (80)	76	84	−452.0 [$\hat{=}$ −20.0%] (332.0)	0 (250.5)	1.561	<0.00001	++ (++)
Hippocampus	78 (78)	75	80	−1115.4 [$\hat{=}$ −17.0%] (817.7)	0 (660.7)	1.519	<0.00001	++ (++)
EntA	78 (78)	76	80	−801.3 [$\hat{=}$ −19.0%] (583.3)	0 (485.3)	1.509	<0.00001	++ (++)
LeftHippocampus	79 (78)	76	81	−588.1 [$\hat{=}$ −18.4%] (423.1)	0 (364.6)	1.502	<0.00001	++ (++)
RightAmygdala	80 (80)	77	83	−232.2 [$\hat{=}$ −20.2%] (182.6)	0 (139.4)	1.452	<0.00001	++ (++)
LeftAmygdala	78 (78)	75	81	−219.8 [$\hat{=}$ −19.7%] (179.7)	0 (135.5)	1.403	<0.00001	++ (++)
RightHippocampus	76 (75)	71	79	−527.3 [$\hat{=}$ −15.7%] (488.0)	0 (339.1)	1.280	<0.00001	++ (++)
InfLatVent	78 (77)	65	89	1330.8 [$\hat{=}$ 65.8%] (1367.4)	0 (702.2)	1.267	<0.00001	++ (++)
LeftInfLatVent	77 (76)	66	86	649.5 [$\hat{=}$ 68.2%] (677.1)	0 (360.6)	1.237	<0.00001	++ (++)
ITG	71 (71)	69	73	−2588.0 [$\hat{=}$ −10.7%] (2506.9)	0 (1954.5)	1.168	<0.00001	++ (++)
**progressive MCI** (**N = 177**, **Positives**^**P**^) **vs**. **stable MCI** (**N = 166**, **Negatives**^**N**^) (**baseline analysis**, ^**†**^**volumes corrected for age/gender/brain size**)								
**structure**	**ACC** (**bACC**)	**SENS**	**SPEC**	**mean [rel**. **to HC]** (**SD**) **[mm**^**3**^**]**^**P**,**†**^	**mean [rel**. **to HC]** (**SD**) **[mm**^**3**^**]**^**N**,**†**^	**effect size** (**d**)	**p-value**	**sig**. (**corr**.)
RandomForest (all features)	68 (68)	72	64					
SVM (all features)	67 (67)	70	64					
(surrogate structures)								
BrainTissue	60 (60)	51	69	−12652.2 [$\hat{=}$ −1.1%] (20144.6)	−4372.1 [$\hat{=}$ −0.4%] (20741.3)	0.405	0.00021	++ (+)
Ventricles	60 (60)	51	70	12483.5 [$\hat{=}$ 35.6%] (20045.3)	4427.7 [$\hat{=}$ 9.7%] (20676.8)	0.396	0.00029	++ (+)
CorticalGreyMatter	56 (56)	55	56	−16743.9 [$\hat{=}$ −3.2%] (27348.8)	−6929.4 [$\hat{=}$ −1.3%] (26948.6)	0.361	0.00091	++ (o)
Brain	51 (51)	52	49	1222424.9 (131084.2)	1238635.5 (118830.2)	0.129	0.23200	o (o)
WhiteMatter	48 (48)	47	49	5966.3 [$\hat{=}$ 1.3%] (27919.1)	4607.3 [$\hat{=}$ 1.0%] (26735.5)	0.050	0.64592	o (o)
DeepGreyMatter	47 (47)	46	48	−1874.6 [$\hat{=}$ −1.0%] (11838.0)	−2050.0 [$\hat{=}$ −1.1%] (11988.8)	0.015	0.89167	o (o)
(selected individual structures)								
Amygdala	65 (65)	63	68	−387.4 [$\hat{=}$ −16.5%] (308.7)	−149.4 [$\hat{=}$ −6.4%] (352.2)	0.720	<0.00001	++ (++)
LeftAmygdala	62 (62)	61	64	−191.6 [$\hat{=}$ −16.7%] (161.2)	−70.4 [$\hat{=}$ −6.0%] (189.7)	0.690	<0.00001	++ (++)
InfLatVent	64 (64)	54	74	1034.0 [$\hat{=}$ 53.8%] (1097.9)	358.6 [$\hat{=}$ 17.4%] (903.1)	0.670	<0.00001	++ (++)
LeftInfLatVent	65 (65)	56	75	480.0 [$\hat{=}$ 53.9%] (538.2)	156.3 [$\hat{=}$ 15.8%] (451.1)	0.650	<0.00001	++ (++)
RightAmygdala	65 (65)	63	67	−195.8 [$\hat{=}$ −16.4%] (176.7)	−79.0 [$\hat{=}$ −6.7%] (184.1)	0.648	<0.00001	++ (++)
EntA	61 (61)	61	60	−678.3 [$\hat{=}$ −15.6%] (565.7)	−294.0 [$\hat{=}$ −6.7%] (622.8)	0.647	<0.00001	++ (++)
RightInfLatVent	64 (64)	54	74	554.0 [$\hat{=}$ 53.9%] (689.1)	202.3 [$\hat{=}$ 18.9%] (527.5)	0.571	<0.00001	++ (++)
Hippocampus	61 (61)	63	60	−1042.8 [$\hat{=}$ −15.6%] (817.1)	−604.7 [$\hat{=}$ −8.9%] (786.1)	0.546	<0.00001	++ (++)
RightHippocampus	63 (63)	61	64	−514.7 [$\hat{=}$ −15.1%] (442.9)	−287.7 [$\hat{=}$ −8.3%] (408.3)	0.532	<0.00001	++ (++)
MTG	60 (60)	59	60	−2305.3 [$\hat{=}$ −7.9%] (2698.1)	−925.8 [$\hat{=}$ −3.0%] (2708.9)	0.510	<0.00001	++ (++)

Individual structures are sorted by effect size. The 10 structures with largest effect size are listed explicitly. Significant group differences indicated by + (p < 0.05) and ++ (p < 0.001). Bonferroni-corrected significance in parentheses. Features were corrected for age, gender and brain size. Mean also shown in % with respect to sample-specific reference volume used for feature correction.

### Longitudinal analysis

**Figure 4:**
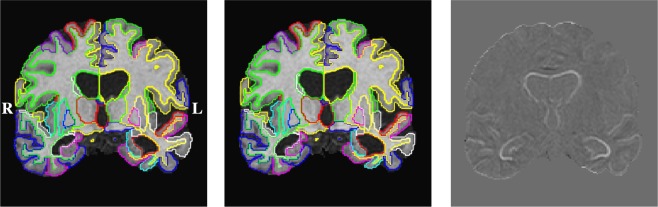
Example longitudinal segmentation results of baseline (left) and month 24 (middle) follow-up images of a patient diagnosed AD (ADNI_018_S_0286) in coronal section. Substantial hippocampal atrophy (measured: −7.81%) and ventricular enlargement (16.5%) are apparent in the difference image after affine registration (right).

In a further longitudinal analysis we investigate the volume change of individual anatomical regions with respect to disease stage. Atrophy was measured for the month-12 (m12) or month-24 (m24) follow-up images with respect to their corresponding baseline (bl) images. In the following, we show atrophy rates with their corresponding sample sizes as well as discriminative power to distinguish between disease stages. Sections from an example segmentation obtained on a subject diagnosed with AD are shown in Fig. 4.

#### Atrophy rates and sample sizes

**Figure 5:**
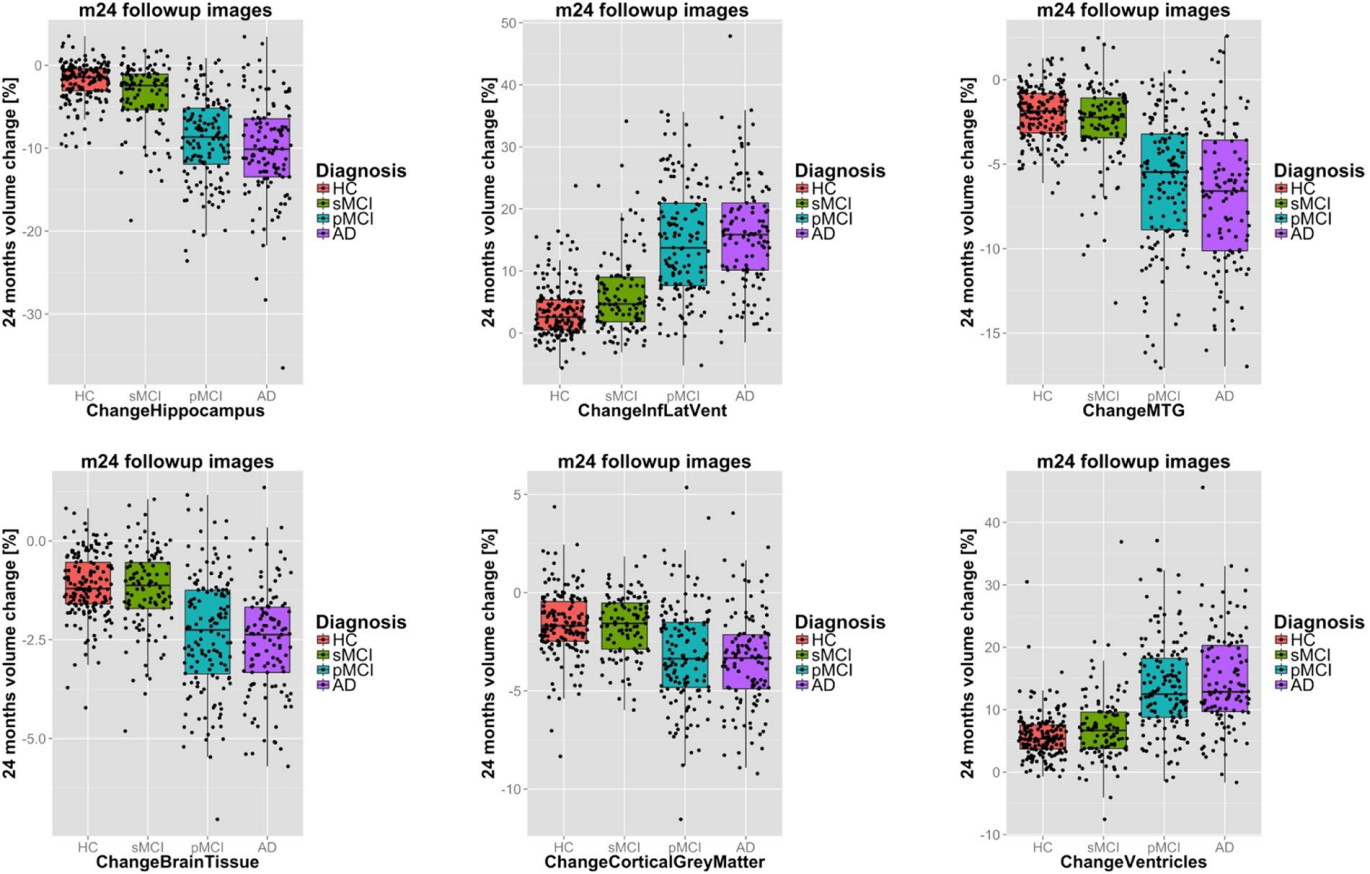
Boxplots of volume changes for selected brain structures (top) and surrogate structures (bottom) from baseline to month 24 follow-up image for different clinical groups. Features selected based on their performance in classifying the investigated disease groups (c.f. Tables 3 and 4).

Atrophy rates for selected structures can be found in Table 2 as well as in Table 3 for AD/HC and in Table 4 for pMCI/sMCI. The distribution of the volume change of six selected structures is shown in Fig. 5 for the four investigated clinical groups (HC, sMCI, pMCI and AD). In AD patients, GM structures such as the hippocampus (HC: −1.1%, AD: −4.8%) or the medial temporal gyrus (HC: −1.1%, AD: −3.8%) are subject to significant volume loss between baseline and m12. Concurrently, ventricles expand rapidly in AD patients (HC: 2.8%, AD: 7.2%). Overall, sMCI subjects show atrophy patterns similar to HC, while atrophy patterns in MCI subjects converting to AD are similar to those in AD patients. The amount of atrophy measured between the baseline and m24 are on the order of twice those measured between baseline and m12. The results indicate that the atrophy rate in the entorhinal region is slightly higher in pMCI subjects than in patients with AD. A volume change rate of −4.0% (±3.7) for pMCI subjects and −3.7% (±4.1) for AD patients was measured at m12. Respectively, a volume change rate of −7.4% (±4.7) for pMCI subjects and −7.0% (±5.5) for AD patients was measured at m24.

**Table 2:** Mean volume change of selected structures in % with corresponding sample sizes for different clinical groups. Standard deviation in parentheses.

bl → m12		hippocampus	inf. lat. vent.	lat. vent.	med. temp. gyr.	brain tissue	ventricles	white matter	cort. GM	deep GM
Atrophy rates	HC	−1.1 (1.7)	1.8 (3.3)	3.0 (3.2)	−1.1 (1.5)	−0.5 (0.8)	2.8 (3.0)	−0.2 (0.7)	−0.8 (1.4)	−0.8 (1.3)
	sMCI	−1.7 (2.2)	2.6 (3.8)	3.6 (3.3)	−1.4 (1.8)	−0.6 (0.8)	3.4 (3.1)	−0.4 (0.7)	−0.8 (1.3)	−0.7 (1.3)
	pMCI	−4.1 (3.2)	6.7 (5.6)	7.1 (4.6)	−3.1 (2.4)	−1.2 (1.0)	6.8 (4.3)	−0.6 (0.9)	−1.8 (1.7)	−1.3 (1.2)
	AD	−4.8 (3.7)	7.5 (5.5)	7.6 (4.9)	−3.8 (2.7)	−1.3 (1.1)	7.2 (4.6)	−0.8 (1.0)	−1.9 (2.3)	−1.4 (1.6)
Sample sizes	sMCI (uncor.)	412	548	217	384	383	215	966	674	739
	sMCI (HC-cor.)	3130	5190	7499	8823	17269	6337	2834	18432936	1429741
	pMCI (uncor.)	155	175	104	143	168	100	488	235	218
	pMCI (HC-cor.)	284	321	309	351	535	285	864	813	1349
	AD (uncor.)	148	134	104	133	182	101	413	366	336
	AD (HC-cor.)	244	228	285	270	493	265	640	640	1133
**bl** → **m24**		**hippocampus**	**inf**. **lat**. **vent**.	**lat**. **vent**.	**med**. **temp**. **gyr**.	**brain tissue**	**ventricles**	**white matter**	**cort**. **GM**	**deep GM**
Atrophy rates	HC	−2.0 (2.3)	3.5 (4.5)	6.3 (4.0)	−2 (1.6)	−1.1 (0.8)	5.8 (3.7)	−0.6 (0.8)	−1.6 (1.7)	−1.3 (1.3)
	sMCI	−3.7 (3.8)	6.2 (6.5)	7.5 (6.0)	−2.6 (2.6)	−1.2 (1.0)	7.1 (5.6)	−0.8 (1.0)	−1.7 (1.5)	−1.2 (1.0)
	pMCI	−8.9 (5.1)	14.6 (8.9)	14.4 (7.9)	−6.1 (3.9)	−2.3 (1.5)	13.7 (7.4)	−1.6 (1.3)	−3.2 (2.5)	−2.1 (1.4)
	AD	−10.2 (6.2)	15.9 (8.8)	15.4 (8.5)	−6.8 (4.1)	−2.6 (1.3)	14.7 (7.8)	−1.8 (1.4)	−3.5 (2.4)	−2.3 (1.8)
Sample sizes	sMCI (uncor.)	264	274	158	246	173	158	370	215	176
	sMCI (HC-cor.)	1166	1446	6179	4588	23154	5166	4764	102542	23243
	pMCI (uncor.)	82	92	76	105	99	73	163	155	107
	pMCI (HC-cor.)	136	160	241	233	372	220	412	618	782
	AD (uncor.)	93	76	75	92	67	71	145	123	162
	AD (HC-cor.)	142	126	217	185	216	195	316	420	980

Measurements based on volume change from baseline to 12 months (top table) or 24 months (bottom table) follow-up visit. Corrected sample sizes were computed on the excess change over normal aging.

**Table 3:** Classification results in % (6-fold cross-validation, LDA 100 runs, RF/SVM 20 runs) for distinguishing between AD and HC based on volume change from baseline to m12 (top) or m24 (bottom). Individual structures are sorted by effect size. The 5 structures with largest effect size are listed explicitly. Significant group differences indicated by + (p < 0.05) and ++ (p < 0.001). Bonferroni-corrected significance in parentheses.

AD patients (N = 195, Positives^P^) vs. Healthy Controls (N = 290, Negatives^N^) (longitudinal analysis, bl → m12)								
structure	ACC (bACC)	SENS	SPEC	mean (SD) [%]^P^	mean (SD) [%]^N^	effect size (d)	p-value	sig. (corr.)
RandomForest (all features)	85 (84)	78	90					
SVM (all features)	84 (82)	71	93					
(surrogate structures)								
Ventricles	75 (74)	64	83	7.2 (4.6)	2.8 (3.0)	1.202	<0.00001	++ (++)
BrainTissue	70 (70)	67	72	−1.3 (1.1)	−0.5 (0.8)	0.862	<0.00001	++ (++)
WhiteMatter	73 (73)	68	77	−0.8 (1.0)	−0.2 (0.7)	0.736	<0.00001	++ (++)
CorticalGreyMatter	65 (64)	62	67	−1.9 (2.3)	−0.8 (1.4)	0.585	<0.00001	++ (++)
Brain	64 (64)	63	65	−0.9 (1.0)	−0.4 (0.7)	0.574	<0.00001	++ (++)
DeepGreyMatter	66 (64)	57	71	−1.4 (1.7)	−0.8 (1.3)	0.455	<0.00001	++ (++)
(selectedindividual structures)								
Hippocampus	80 (78)	67	88	−4.8 (3.7)	−1.1 (1.7)	1.400	<0.00001	++ (++)
InfLatVent	79 (77)	69	86	7.5 (5.5)	1.8 (3.3)	1.334	<0.00001	++ (++)
LeftHippocampus	80 (78)	70	87	−4.9 (4.2)	−1.1 (1.7)	1.287	<0.00001	++ (++)
MTG	76 (74)	67	82	−3.8 (2.8)	−1.1 (1.5)	1.274	<0.00001	++ (++)
RightInfLatVent	77 (75)	65	84	7.4 (6.4)	1.8 (3.3)	1.185	<0.00001	++ (++)
**AD patients** (**N = 117**, **Positives**^**P**^) **vs**. **Healthy Controls** (**N = 168**, **Negatives**^**N**^) (**longitudinal analysis**, **bl → m24**)								
RandomForest (all features)	89 (88)	81	94					
SVM (all features)	89 (88)	80	95					
(surrogate structures)								
Ventricles	83 (81)	70	92	14.7 (7.9)	5.8 (3.7)	1.531	<0.00001	++ (++)
BrainTissue	80 (78)	71	86	−2.6 (1.3)	−1.1 (0.8)	1.353	<0.00001	++ (++)
WhiteMatter	75 (74)	65	82	−1.8 (1.4)	−0.6 (0.8)	1.121	<0.00001	++ (++)
CorticalGreyMatter	73 (73)	69	77	−3.5 (2.4)	−1.6 (1.7)	0.928	<0.00001	++ (++)
Brain	68 (67)	65	70	−1.5 (1.0)	−0.8 (0.7)	0.847	<0.00001	++ (++)
DeepGreyMatter	67 (66)	63	69	−2.3 (1.8)	−1.3 (1.3)	0.604	<0.00001	++ (++)
(selected individual structures)								
Hippocampus	87 (85)	77	94	−10.2 (6.2)	−2.0 (2.4)	1.880	<0.00001	++ (++)
InfLatVent	84 (83)	76	90	15.9 (8.8)	3.5 (4.5)	1.871	<0.00001	++ (++)
RightInfLatVent	83 (82)	73	90	16.1 (9.7)	3.5 (4.9)	1.728	<0.00001	++ (++)
LeftHippocampus	86 (84)	77	92	−9.9 (6.6)	−1.9 (2.7)	1.701	<0.00001	++ (++)
LeftInfLatVent	80 (78)	69	88	15.5 (9.5)	3.5 (5.2)	1.652	<0.00001	++ (++)

**Table 4:** Classification results in % (6-fold cross-validation, LDA 100 runs, RF/SVM 20 runs) for distinguishing between pMCI and sMCI based on volume change from baseline to m12 (top) or m24 (bottom). Individual structures are sorted by effect size. The 5 structures with largest effect size are listed explicitly. Significant group differences indicated by + (p < 0.05) and ++ (p < 0.001). Bonferroni-corrected significance in parentheses.

progressive MCI (N = 168, Positives^P^) vs. stable MCI (N = 149, Negatives^N^) (longitudinal analysis, bl → m12)								
structure	ACC (bACC)	SENS	SPEC	mean (SD) [%]^P^	mean (SD) [%]^N^	effect size (d)	p-value	sig. (corr.)
RandomForest (all features)	74 (73)	77	70					
SVM (all features)	74 (74)	72	75					
(surrogate structures)								
Ventricles	68 (69)	63	74	6.8 (4.3)	3.4 (3.1)	0.890	<0.00001	++ (++)
BrainTissue	63 (63)	64	63	−1.2 (1.0)	−0.6 (0.8)	0.652	<0.00001	++ (++)
CorticalGreyMatter	64 (64)	67	61	−1.8 (1.7)	−0.8 (1.3)	0.608	<0.00001	++ (++)
Brain	58 (58)	62	54	−0.8 (0.8)	−0.4 (0.6)	0.495	0.00002	++ (+)
DeepGreyMatter	64 (64)	62	66	−1.3 (1.2)	−0.7 (1.3)	0.428	0.00017	++ (+)
WhiteMatter	63 (63)	61	64	−0.6 (0.9)	−0.4 (0.7)	0.310	0.00615	+ (o)
(selected individual structures)								
Hippocampus	67 (67)	59	76	−4.1 (3.2)	−1.7 (2.2)	0.867	<0.00001	++ (++)
LateralVentricle	67 (68)	61	75	7.1 (4.6)	3.6 (3.4)	0.866	<0.00001	++ (++)
InfLatVent	69 (69)	63	75	6.7 (5.6)	2.6 (3.8)	0.845	<0.00001	++ (++)
LeftHippocampus	67 (68)	60	75	−4.2 (3.3)	−1.7 (2.5)	0.845	<0.00001	++ (++)
LeftLateralVentricle	66 (67)	60	73	7.2 (4.8)	3.6 (3.5)	0.836	<0.00001	++ (++)
**progressive MCI (N = 140, Positives** ^**P**^ **) vs. stable MCI (N = 107, Negatives** ^**N**^ **) (longitudinal analysis, bl → m24)**								
RandomForest (all features)	79 (78)	82	74					
SVM (all features)	76 (76)	76	76					
(surrogate structures)								
Ventricles	71 (72)	65	80	13.7 (7.4)	7.1 (5.6)	0.989	<0.00001	++ (++)
BrainTissue	68 (69)	62	76	−2.3 (1.5)	−1.2 (1.0)	0.845	<0.00001	++ (++)
DeepGreyMatter	66 (67)	64	69	−2.1 (1.4)	−1.2 (1.0)	0.714	<0.00001	++ (++)
CorticalGreyMatter	65 (66)	61	71	−3.2 (2.5)	−1.7 (1.6)	0.707	<0.00001	++ (++)
Brain	63 (63)	60	67	−1.5 (1.1)	−0.8 (0.7)	0.707	<0.00001	++ (++)
WhiteMatter	64 (65)	55	74	−1.6 (1.3)	−0.8 (1.0)	0.661	<0.00001	++ (++)
(selected individual structures)								
FuG	75 (76)	69	83	−3.1 (2.1)	−1.0 (1.4)	1.141	<0.00001	++ (++)
Hippocampus	73 (74)	68	80	−8.9 (5.1)	−3.7 (3.8)	1.118	<0.00001	++ (++)
LeftHippocampus	74 (74)	72	77	−9.3 (5.6)	−3.8 (4.2)	1.083	<0.00001	++ (++)
EntA	71 (72)	66	77	−7.4 (4.7)	−2.8 (3.6)	1.070	<0.00001	++ (++)
InfLatVent	70 (72)	62	81	14.6 (8.9)	6.2 (6.6)	1.053	<0.00001	++ (++)

Based on the atrophy rates, sample sizes were calculated to detect a 25% change in atrophy rate with 80% power at a 5% significance level. The atrophy rates and sample sizes for selected structures are shown in Table 2. The smallest sample sizes (corrected for normal aging) were computed for the inferior lateral ventricles with 228 subjects for bl  → m12 and 126 subjects for bl → m24. The measured atrophy rates of other structures such as the hippocampus or the medial temporal gyrus yielded sample sizes at a similar level. Atrophy rates for all investigated structures can be found in the supplementary material.

#### Disease classification using structural volume change

We further investigated the potential of structural atrophy rates to distinguish between the clinical groups AD vs. HC and pMCI vs. sMCI. P-values, effect sizes and classification accuracies were calculated to quantify group separation. The results are shown in Table 3 (AD vs. HC) and Table 4 (pMCI vs. sMCI).

The most discriminative structure to distinguish between AD and HC was the hippocampus (d_m12_ = 1.40, d_m24_ = 1.88). Based on m12 atrophy a bACC_m12_ of 78% (SENS: 67%, SPEC: 88%) was calculated, for m24 atrophy a balanced accuracy of 85% respectively. Combining all derived atrophy features in a RF classifier substantially increased classification results to bACC_m12_: 84%; bACC_m24_: 88%. Hippocampal atrophy was also a very good feature for classifying progressive versus stable MCI subjects: bACC_m12_: 67%; bACC_m24_: 74%. However, the highest individual classification accuracy was obtained at m12 for the medial temporal gyrus (bACC_m12_: 70%, SENS: 65%, SPEC: 75%) and the inferior lateral ventricles (bACC_m12_: 69%, SENS: 63%, SPEC: 75%). Over 24 months, atrophy in the fusiform gyrus was most informative for classifying MCI subjects (bACC_m24_: 76%). Exploiting all available longitudinal features in an RF classifier increased MCI classification accuracy to bACC_m12_: 73%; bACC_m24_: 78%. Ventricular enlargement is more discriminative than a reduction in brain tissue for both AD versus HC and pMCI versus sMCI classification. An overview over all considered features and their individual classification performance can be found in the supplementary material.

## Discussion

In this study, sets of 1069 baseline, 802 m12, and 532 m24 follow-up images from the ADNI-1/-GO/-2 cohort were analyzed. Considering the size and heterogeneity of the database, we expect our findings to be applicable to other cohorts.

A selection of articles is listed in Table 5 to present our classification results in the context of those reported in the literature. When comparing results to other studies it must be noted that these did not use identical data subsets from the ADNI cohorts. A further potential confounding factor is the definition of the sMCI and pMCI disease groups. In our study, the stratification of MCI subjects in sMCI and pMCI is well-defined. However, this definition is not identical across published studies.

**Table 5:** Overview over selected articles that use features from T1w MR images from the ADNI cohort. Table adapted from Falahati *et al*.^7^. CTH: cortical thickness, ENR: elastic net regression, HV: hippocampus, LLE: locally linear embedding, MBL: manifold-based learning, MIL: multiple instance learning, OPLS: orthogonal partial least square to latent structure, SR: spare regression, TBM: tensor-based morphometry.

Article	Dataset (field strength)	Features	Classifier	AD vs. HC				pMCI vs. sMCI			
				bACC	SENS	SPEC	N_AD_/N_HC_	bACC	SENS	SPEC	N_pMCI_/N_sMCI_
MALPEM	ADNI1/Go/2 (1.5/3 T)	ROI volumes	RF	86	83	90	322/404	68	72	64	177/166
MALPEM	ADNI1/Go/2 (1.5/3 T)	ROI volumes	SVM	89	86	92	322/404	67	70	64	177/166
Beheshti *et al*.^82^	ADNI1 (3T)	VBM+ROM and intensity features	SVM	93	89.1	96.8	92/94	75	76.9	73.2	71/65
Coupe *et al*.^28^	ADNI1 (1.5T)	ROI volumes and grading features	LDA	90.5	87	94	198/231	73.5	73	74	167/238
Chincarini *et al*.^63^	ADNI1 (1.5T)	intensity and textural features features from 9 ROIs	SVM	91.5	89	94	144/189	68.5	72	65	136/166
Guerr`ero *et al*.^49^	ADNI1 (1.5T)	learned ROIs via SR + MBL	SVM	85.5	86	85	106/175	71	75	67	116/114
Hu *et al*.^83^	ADNI1 (1.5T)	VBM and wavelet frame features	SVM	84.1	82.5	85.6	288/188	76.7	71.8	82.3	71/62
Liu *et al*.^61^	ADNI1 (1.5T)	ROI volumes, CTH	ENR + LLE	89.5	86	93	86/137	68	80	56	97/93
Tong *et al*.^50^	ADNI1 (1.5T)	local intensity patches	MIL	89	85	93	198/231	70	67	73	167/238
Wee *et al*.^60^	ADNI1 (1.5T)	correlative and ROI-based morphological features	SVM	92.4	90.4	94.3	198/200	74.0	63.5	84.4	89/111
Westman *et al*.^62^	ADNI1 (1.5T)	ROI volumes, CTH, curvature and folding features	OPLS	91.5	90	93	187/225	71.2	75.9	66.5	87/200
Wolz *et al.*^34^	ADNI1 (1.5T)	HV, CTH, TBM, MBL	LDA	89	93	85	198/231	68	67	69	167/238
Zu *et al*.^84^	ADNI1 (1.5T+PET)	ROI volumes	SVM	96	95.1	94.5	51/52	69.8	66.7	71.4	56/43

Our cross-sectional classification results are very similar to those presented in Wolz *et al*.^34^, which are based on the ADNI-1 cohort. Wolz *et al*.^34^ classified AD vs. HC (bACC: 89%, SENS: 93%, SPEC: 85%) and pMCI vs. sMCI (bACC: 68%, SENS: 67%, SPEC: 69%) based on a multitude of features, including more abstract criteria derived from TBM and manifold-learning based methods. Other studies report even higher classification results of up to bACC: 92.4% for AD vs. HC and bACC: 74.0% for pMCI vs. sMCI classification^60^. Unlike our study, most studies shown in Table 5 analyzed the 1.5 Tesla (T) images of ADNI-1 only. Also usually more complex features such as cortical features^34,60–62^, textural features^63^, manifold-based features^34,61^ or grading based features^28^ are employed. In summary, the results presented in this study are comparable to the state of the art. This is encouraging, as we analyzed a large and heterogeneous dataset acquired at both 1.5T and 3T using structural volumes only, which are features with clear biological interpretations. Furthermore, structures that were found to be most discriminative agree well with those highlighted in Fennema *et al*.^2^. Examples are the hippocampus, amygdala, entorhinal area, and regions within the temporal gyrus in general.

Based on hippocampal atrophy alone a bACC_m12_ of 78% and bACC_m24_ of 85% was calculated for classifying AD vs. HC. These results are similar to those obtained on a different ADNI subset with a method dedicated to hippocampal atrophy measurement^22^: bACC_m12_ of 82% (SENS: 81%, SPEC: 83%); bACC_m24_ of 86% (SENS: 85%, SPEC: 87%). The results for AD vs. HC classification using all longitudinal features are also on par with those using all structural volumes at baseline. However, classification accuracies substantially above 90% were not expected due to potential bias in the study data caused, for example, by diagnostic misclassification, variations in scanner type and field strength, as well as possible remnant differences between participating centres that even strict protocols such as ADNI’s cannot prevent. Using longitudinal information available at the month 24 follow-up visit increased accuracy for classifying sMCI vs. pMCI groups from 68% at baseline to 78%. This confirms the discriminative value of higher structural atrophy rates in MCI subjects who progress to AD. Unlike in patients with AD, atrophy in MCI subjects has not yet manifested itself in substantially reduced structural volumes at baseline.

In Barnes *et al*.^64^ the authors concluded in a large meta-analysis that the annualized hippocampal volume change of healthy elderly people is −1.4% compared with −4.6% for patients with AD. Our results are similar: −1.1% (±1.7) for HC and −4.8% (±3.7) for AD subjects. A mean change of −0.5% (±0.8) and −1.3% (±1.1) from bl to m12 was observed for HC and AD groups for brain tissue. These results are also in line with previously published annual brain volume change rates of around −0.6% for controls and −1.5% for AD patients^65^. This confirms that the employed methodology yields realistic atrophy measurements on individual structures, while providing a comprehensive overview of structural change throughout the whole brain.

For the four structures hippocampus, amygdala, inferior lateral ventricle, and lateral ventricle, their respective counterparts in the left/right brain hemisphere were analyzed separately. The results suggest that structural change in the left hippocampus is slightly more discriminative than change in the right hippocampus. There is no consistent trend for the other investigated structures. In general, features of left-right paired structures perform similarly for all investigated structures and combining them seems, in summary, beneficial.

The use of either SVM or RF classifiers provided substantial improvements over the results obtained using individual features only. Overall SVM and RF performed similarly, with slight advantages for one or the other in individual experiments.

Our experiments confirm that MALPEM is an accurate and sensitive approach for brain image analysis. One of MALPEM’s main advantages is that it delivers a full morphometric analysis of all of 138 structures, unlike specialized methods that only work on a small selected set of individual structures. Another important strength is that our methods allow both the accurate analysis of single images (MALPEM) as well as image series (MALPEM4D). Thus, the presented methodology has strong potential to support both cross-sectional and longitudinal studies that include MR imaging of the brain.

As part of this work, we created a morphometry database of unprecedented size and accuracy, which we share with the community. This database provides pincram brain extractions and MALPEM segmentations of 5074 MR images, as well as longitudinal features extracted from 1334 MR image series. In previous work, we shared a database built on images from ADNI-1^66^. The present development differs from this past effort in important ways that reflect developments of ADNI (data from ADNI-2 and ADNI-GO became available in the meantime) as well as software improvements (MALPEM yields more accurate segmentations than the MAPER method^67^ used in the previous project). The previous database was substantially smaller and contained only cross-sectional data (996 baseline and screening images). Another difference is in the atlas database chosen: compared to the atlases used for the previous resource^68,69^, the NMM atlases offer more detailed cortical subdivisions.

We anticipate that our new morphometry database will be an immensely valuable resource for future research on classification and modeling approaches. It can further enable the optimization of training-data intense deep learning methodologies.

## Methods

### Materials

For this study a subset of T1-weighted (T1w) MR brain images was analyzed from all studies by the Alzheimer’s Disease Neuroimaging Initiative (ADNI) for which data are currently available (ADNI-1/-GO/-2). The ADNI was launched in 2003 as a public-private partnership, led by Principal Investigator Michael W. Weiner, MD. ADNI enrols participants between the ages of 55 and 90 who are recruited at 57 sites in the United States and Canada. After obtaining informed consent, participants undergo a series of initial tests that are repeated at intervals over subsequent years, including a clinical evaluation, neuropsychological tests, genetic testing, lumbar puncture, and MRI and PET scans. Participants were studied under ADNI protocols that were approved by the Institutional Review Board (IRB) at each recruitment site. A listing of sites with named Site Investigators can be found online at https://adni.loni.usc.edu/wp-content/uploads/how_to_apply/ADNI_Acknowledgement_List.pdf (last accessed 30 June 2018). ADNI procedures manuals were developed as a resource for ADNI research sites. They are tailored to site Study Coordinator and support staff. The ADNI MRI Technical Procedures manuals are developed by the ADNI Imaging Core to direct MRI technicians in the scanning of ADNI subjects. For further details and up-to-date information please refer to the supplementary material and http://www.adni-info.org (last accessed 15 March 2018). All methods described in this study were performed in accordance with relevant guidelines and regulations. When the present study was started, we retrieved clinical information and corresponding MR images based on the ADNIMERGE package. Specifically, 5074 (1674 baseline, 3400 follow-up) images were processed with MALPEM. Subjects had been scanned up to 10 times, with the last follow-up image acquired 8 years after the baseline. For the present analysis, a subset of these 5074 processed images was selected based on clinical information. We applied the following criteria for inclusion/exclusion of individual subjects:

- All subjects who reverted at any time point from a more severe to a less severe disease stage, i.e. AD → MCI or MCI → HC, were excluded from both the cross-sectional (N = 68) and the longitudinal analysis.
- All subjects with baseline diagnosis early MCI (N = 277) or SMC (N = 76) were excluded from both the cross-sectional and the longitudinal analysis.
- The *sMCI* group was defined as those subjects who were diagnosed as MCI (called ‘late MCI’ in ADNI-GO/-2) at baseline and remained at the MCI stage for at least two years and until the most recent diagnosis which was available. This means that subjects for whom this information was not available, e.g. because the repository did not contain a corresponding m24 or later visit, were excluded (N = 130).
- The *pMCI* group was defined as those subjects who were diagnosed as MCI at baseline and converted within two years follow-up to a diagnosis of probable AD.
- Subjects who were diagnosed as MCI at baseline but converted to probable AD more than two years later (N = 54) were excluded from the analyses and neither considered as sMCI nor pMCI.
- The m12 image (I373205) of one subject (ADNI_007_S_4568) was reviewed after irregular volume measurements and excluded manually from the analysis due to poor image quality.
- All subjects listed in Table 6 fulfil the above criteria. For the longitudinal analysis, however, all subjects that converted at any time of the study from HC to a symptomatic stage (e.g. to MCI or even to AD) were excluded (N = 52).

Table 6 gives an overview over the baseline images considered in the conducted analyses. Note that this is a well-defined subset of all 5074 images processed. Lists of the processed filenames that also include the unique image identifier are available online at 10.12751/g-node.aa605a^70^.

**Table 6:** Overview of the analyzed subjects from the ADNI cohort, including age and clinical information at baseline.

	ALL	HC	sMCI	pMCI	AD
# of subjects/images at baseline	1069	404	166	177	322
gender (# male/# female)	581/488	202/202	98/68	104/73	177/145
years of age (median [min; max])	74.6 [48.1; 91.4]	74.2 [59.8; 89.6]	74.4 [55.9; 91.4]	74.3 [48.1; 88.3]	75.8 [55.1; 91.4]
ApoE4 (# 0/# 1/# 2)^†^	547/407/113	293/101/9	93/60/13	57/91/29	104/155/62
MMSE (median [min; max])	27 [18; 30]	29 [24; 30]	28 [24; 30]	26 [23; 30]	23 [18; 27]
FAQ (median [min; max])^‡^	1 [0; 30]	0 [0; 6]	1 [0; 21]	5 [0; 21]	13 [0; 30]
CDRSB (median [min; max])	1.5 [0; 10]	0 [0; 1]	1.5 [0.5; 4]	2 [0.5; 5]	4.5 [1; 10]
FieldStrength (1.5T/3T)	653/416	223/181	112/54	129/48	189/133
# of subjects/images at month 12	802	195	149	168	290
# of subjects/images at month 24	532	168	107	140	117

^†^Not available for 2 subjects, ^‡^not available for 1 subject.

### Preprocessing

As preprocessed versions of the images were downloaded from ADNI, no additional preprocessing was performed^71^. Brain masks were calculated for all available baseline images using pincram^57^. Brain masks were visually reviewed and some were recalculated with an updated pincram atlas database. Follow-up images were brain-extracted utilizing the corresponding baseline brain masks, which were transformed using rigid intrasubject registration.

### Cross-sectional and longitudinal segmentation

All 5074 baseline and follow-up images were segmented individually using MALPEM as described in Ledig *et al*.^53^. As the atlas database, we used the manually annotated Neuromorphometrics (NMM) brain atlases (n = 30; provided by Neuromorphometrics, Inc. under academic subscription, http://Neuromorphometrics.com/, last accessed 15 March 2018). The atlas label sets contain expert delineations of 40 non-cortical and 98 cortical brain regions. A description of the individual structures is provided in the supplementary material. MALPEM was recently validated in an independent study led by Johnson *et al*.^54^ where the authors compared state-of-the-art segmentation methods in the context of Huntington’s disease. The refined, time-point specific probabilistic segmentation output and the intensity-normalized, brain-extracted images of MALPEM are then employed to perform the consistent longitudinal segmentation as described in Ledig *et al*.^35^ (MALPEM4D). MALPEM4D is an approach that employs spatially and temporally varying coupling weights between time points to obtain temporally consistent segmentation estimates. In this work, MALPEM4D incorporates symmetric affine intra-subject registration^72,73^ and corrects for differential bias between intra-subject acquisitions using unweighted differential bias correction^74^. MALPEM4D is run on pairs of images to separately estimate volume changes bl → m12 and bl → m24.

### Features and classification

For the cross-sectional analysis at baseline, structural volumes were calculated with MALPEM and not MALPEM4D, as the latter exploits information of later scanning time points, which was not available at baseline. For the longitudinal analysis structural volumes were extracted based on the respective MALPEM4D segmentations.

As features, all available structural volumes were employed. For paired structures, the left and right volumes were merged (98/2 = 49 cortical plus 28/2 + 7 = 21 non-cortical features). Note that seven non-cortical structures are unpaired (3rd ventricle, 4th ventricle, brainstem, CSF, cerebellar vermal lobules I–V, cerebellar vermal lobules VI–VII, and cerebellar vermal lobules VIII–X). Exceptions were made for the amygdala, hippocampus, inferior lateral ventricles, and lateral ventricles. As it is expected that these structures are particularly informative, their left and right volumes were retained as separate features (8 features). This allowed us to investigate asymmetric involvement of these structures in the disease progression. Individual structures were further summarized as ventricles, cortical grey matter, deep grey matter, white matter, brain tissue and total brain volume (brain tissue including ventricles/CSF) (6 features). In total 86 features were considered, including age and gender.

For classification, a 6-fold cross-validation (CV) was performed using an linear discriminant analysis (LDA) classifier for individual features. When combining multiple features, both SVM and RF classifiers were employed. A classification framework was implemented using MATLAB (The MathWorks Inc, Natick, MA, USA) that relies on classify (LDA), TreeBagger (RF, 100 trees) and libSVM (linear SVM^75^). Features were normalized (rescaled) individually to a range from 0 to 1 for the SVM classification. In addition to the standard classification accuracy (ACC), we also quantified the balanced classification accuracy (bACC^76^) to account for imbalanced group sizes. The bACC is calculated as the arithmetic mean of SENS and SPEC.

Significance levels were quantified as p-values of two-sided, unpaired Student’s t-tests. We employed the conservative Bonferroni correction to correct for multiple comparisons. Further, effect sizes were calculated as Cohen’s d by dividing the differences of the sample means (absolute value) by their pooled standard deviation^2,77,78^. According to Cohen^77^ an effect size of d = 0.2 can be considered as small, of d = 0.5 as medium and of d = 0.8 as large. Reporting the effect size in addition to the p-value is important as it quantifies the magnitude of a group difference, while a low p-value by itself only confirms its existence^79^.

### Correction for nuisance factors

**Figure 6:**
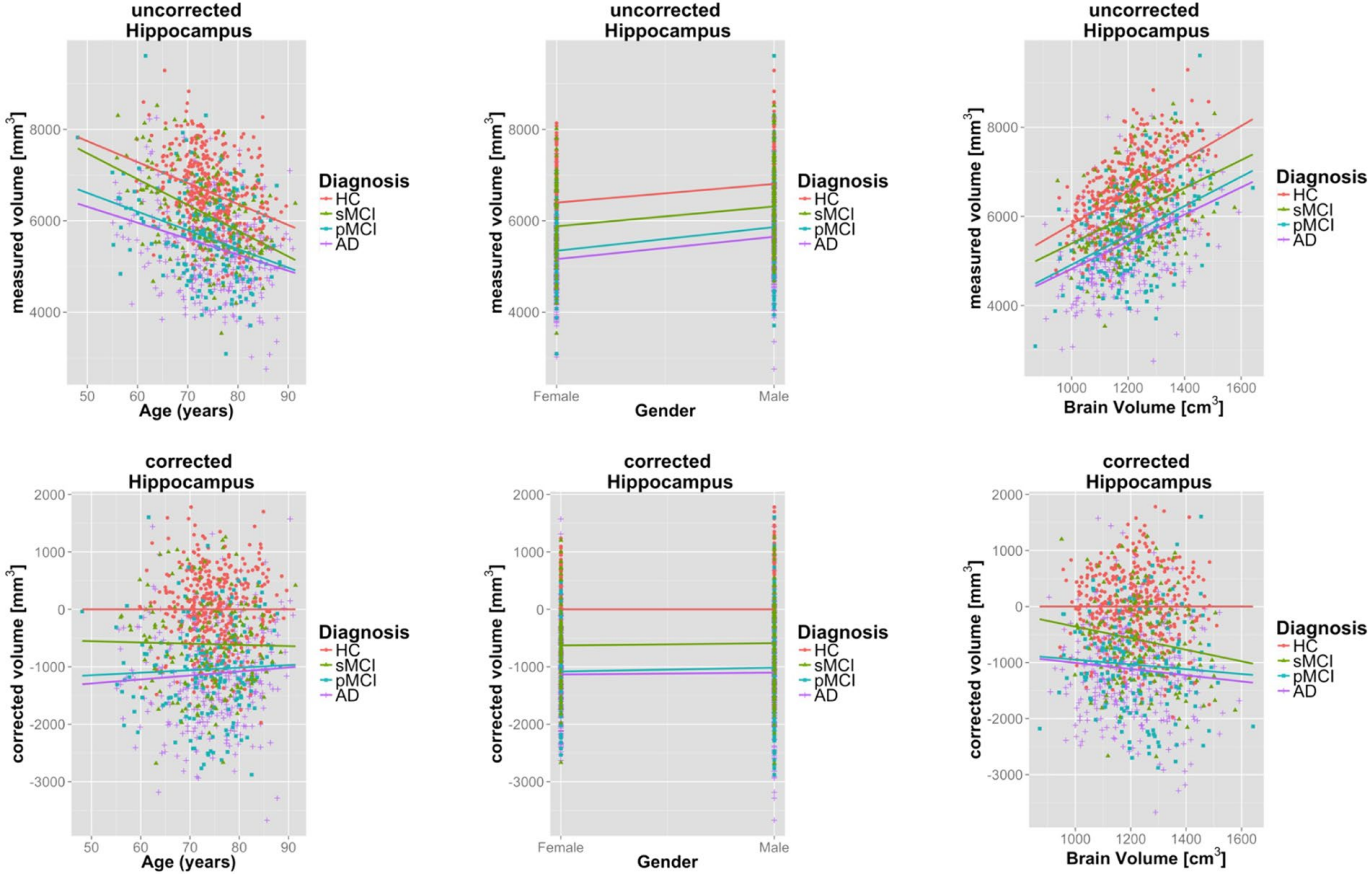
Top: Dependence of hippocampal volume on age (left), gender (middle) and brain volume (right). Bottom: Corresponding s corrected for nuisance factors age, gender and brain size. Overlaid regression lines for distinct disease groups with corresponding regression lines.

The volume of many individual brain structures diminishes during the course of normal, healthy aging. Also, strong correlations between structural volumes and overall head size are well established^80^. This is illustrated in Fig. 6, where linear regressors are fit to hippocampal volumes with respect to age, gender, and brain volume based on the processed HC subjects.

We followed a multiple linear regression approach as described in Koikkalainen *et al*.^81^ and investigated the nuisance factors *age*, *gender* and *brain* size. Specifically, a separate linear regressor was fit using the processed healthy control subjects for each individual structure and the abovementioned predictor variables. Each multivariate regressor is defined by the slope for each predictor *c*_age_, *c*_gender_ and *c*_size_ and an intercept *b*. Assuming *M* distinct features, feature *m* of subject *n*, denoted by ${{F}_{n}}^{m}$, is corrected as:

1 $${{\mathop{F}\limits^{ \sim }}_{n}}^{m}={{F}_{n}}^{m}-({c}_{{\rm{a}}{\rm{g}}{\rm{e}}}^{m}{{\rm{a}}{\rm{g}}{\rm{e}}}_{n}+{c}_{{\rm{g}}{\rm{e}}{\rm{n}}{\rm{d}}{\rm{e}}{\rm{r}}}^{m}{{\rm{g}}{\rm{e}}{\rm{n}}{\rm{d}}{\rm{e}}{\rm{r}}}_{n}+{c}_{{\rm{s}}{\rm{i}}{\rm{z}}{\rm{e}}}^{m}{{\rm{s}}{\rm{i}}{\rm{z}}{\rm{e}}}_{n}+{b}^{m}).$$

To correct for head size, the total brain size (sum of all structures) was used as an approximation of the intracranial volume. This is a commonly used approximation^80^.

The effect of correcting for the nuisance factors patient age, gender and head size is shown in Table 7. The correction substantially increased classification accuracies and effect sizes obtained on individual structures. The benefit of the correction for all investigated structures, including effect sizes, can be found in the supplementary material.

**Table 7:** Balanced classification accuracies in % for distinguishing between HC and AD subjects (effect sizes in parentheses) after correcting for various nuisance factors (100 runs, 6-fold cross-validation, LDA). Largest effect size in bold.

structures/correction	none	age	brain size	gender	all
Ventricles	65 (0.70)	65 (0.72)	68 (0.86)	65 (0.71)	**71** (**0**.**90**)
CorticalGreyMatter	61 (0.53)	62 (0.53)	67 (0.86)	62 (0.67)	**67** (**0**.**90**)
amygdala	75 (1.35)	76 (1.40)	79 (1.48)	77 (1.42)	**80** (**1**.**56**)
hippocampus	75 (1.33)	75 (1.38)	77 (1.45)	76 (1.40)	**78** (**1**.**52**)
EntA	73 (1.28)	74 (1.33)	75 (1.39)	76 (1.41)	**78** (**1**.**51**)
InfLatVent	72 (1.10)	72 (1.16)	73 (1.19)	72 (1.14)	**77** (**1**.**27**)
ITG	66 (0.89)	66 (0.89)	71 (1.13)	71 (1.07)	**71** (**1**.**17**)
MTG	64 (0.74)	63 (0.73)	**69** (**0**.**97**)	66 (0.86)	**69** (**0**.**97**)

The observed benefit of correcting for these confounding factors is in agreement with the literature^81^. In the conducted experiment, correcting for brain size had the biggest effect and correcting for gender had a stronger impact than correcting for age. Correcting for all three nuisance factors (age, gender and brain size) was most beneficial in terms of both classification accuracy and effect size.

An illustration of the volumes before and after correction is provided in Fig. 6. Healthy control subjects have zero mean after correction, and the overall dependence on nuisance factors is clearly reduced.

The corrected volumes were used for the cross-sectional analysis. This means that feature values are no longer actual volumes, but rather volume differences with respect to a healthy population of matched age, gender and brain size. Note that the three independent variables patient age, gender, and head size were not corrected for.

### Calculation of atrophy rates and sample sizes

For a volume $${v}_{{t}^{1}}$$ at baseline and a volume $${v}_{{t}^{2}}$$ at a follow-up time point we calculated *atrophy rates* using the logarithmic transform as $${{\rm{\Delta }}}_{v}({t}^{1},{t}^{2})={{\rm{\Delta }}}_{v}^{\mathrm{log}}({t}^{1},{t}^{2})=$$$$\mathrm{ln}({v}_{{t}^{2}}/{v}_{{t}^{1}})\cdot \mathrm{100 \% }$$. Note that atrophy rate and volume change is used interchangeably, which means that a *positive* atrophy rate indicates an *increase* in volume.

For a power (1 − β) and significance level *α* the *sample size* can be calculated^23^ as:

2 $$N={({z}_{1-{\rm{\beta }}}+{z}_{1-{\rm{\alpha }}\mathrm{/2}})}^{2}\cdot \frac{\mathrm{(2}{{\rm{\sigma }}}_{{\rm{g}}}^{2})}{{{\rm{\Delta }}}^{2}}.$$

Here Δ is the difference in atrophy rate that is to be shown between the clinical groups. In this study sample sizes were calculated to detect a 25% change in atrophy rate (Δ = 0.25 *μ*_g_) with 80% power (*z*_0.8_ ≈ 0.84) at a 5% significance level (*z*_1−0.05/2_ ≈ 1.96). These parameter choices are commonly found in the literature^22,23,72^. It is important to relate atrophy rates in dementia to normal atrophy during aging, as in the uncorrected case it is assumed that 100% treatment effect would effectively reduce the structural atrophy to zero^23^. Sample sizes were thus corrected for normal ageing by evaluating Equation 2 with Δ = 0.25(*μ*_g_−*μ*_healthy_) to reduce the maximal treatment effect to the level of normal ageing. In Equation 2 it is assumed that measurements of healthy atrophy have the same variance as measurements of diseased subjects (σ_g_ ≈ σ_healthy_)^23^. This usually leads to a more conservative estimate.

## Data availability

The datasets generated during and/or analyzed in this study are available online (all resources last accessed 15 March 2018).

- The source code of pincram^57^ and the MALPEM framework^53^ is available at https://github.com/ledigchr/MALPEM, the MIRTK source for the involved binaries is available at https://github.com/BioMedIA/MIRTK.
- The binary brain masks (pincram) and structural segmentations (MALPEM) for 5074 images from the ADNI cohort are available for download at 10.12751/g-node.aa605a^70^.
- All extracted features and selected clinical information (e.g. disease labels) are also available at 10.12751/g-node.aa605a^70^.

## Electronic supplementary material

Supplementary Material
