# Supplementary material for: Structural brain imaging in Alzheimer’s disease and mild cognitive impairment: biomarker analysis and shared morphometry database
Source: Sci Rep. 2018 Jul 26;8:11258. doi: 10.1038/s41598-018-29295-9 (PMC6062561; doi:10.1038/s41598-018-29295-9)
Supplement: Supplementary file 1 — Supplementary Material [file 41598_2018_29295_MOESM1_ESM.pdf]

Supplementary Material - Structural brain imaging in Alzheimer's disease and mild cognitive impairment: biomarker analysis and shared morphometry database

Christian Ledig[1,*], Andreas Schuh[1], Ricardo Guerrero[1], Rolf A. Heckemann[2,3,4], Daniel Rueckert[1]

1 Imperial College London, Department of Computing, London, SW7 2AZ, UK
2 MedTech West, Sahlgrenska University Hospital, 413 45 Gothenburg, Sweden
3 Department of Radiation Therapy, Sahlgrenska Academy, University of Gothenburg, Gothenburg, Sweden
4 Division of Brain Sciences, Imperial College London, London
∗ ledig.christian@gmail.com

Alzheimer's Disease Neuroimaging Initiative (ADNI)

Data used in preparation of this article were obtained from the Alzheimer's Disease Neuroimaging Initiative (ADNI) database (adni.loni.usc.edu). As such, the investigators within the ADNI contributed to the design and implementation of ADNI and/or provided data but did not participate in analysis or writing of this report. A complete listing of ADNI investigators can be found at: http://adni.loni.usc.edu/wp-content/uploads/how_to_apply/ADNI_Acknowledgement_List.pdf.

The ADNI was launched in 2003 as a public-private partnership, led by Principal Investigator Michael W. Weiner, MD. The primary goal of ADNI has been to test whether serial magnetic resonance imaging (MRI), positron emission tomography (PET), other biological markers, and clinical and neuropsychological assessment can be combined to measure the progression of mild cognitive impairment (MCI) and early Alzheimer's disease (AD). For up-to-date information, see http://www.adni-info.org (last accessed 15 March 2018). ADNI is a multi-center study with over 50 acquisition sites in North America. The first ADNI study, ADNI-1, was subsequently continued as ADNI-GO and ADNI-2, with a further continuation into ADNI-3 currently in progress. Throughout the three studies considered here more than 1500 participants were enrolled including healthy control subjects and subjects diagnosed with different stages of MCI or AD.

Anatomical regions as defined in the NMM atlas

Table 1. Structure names of non-cortical structures in the NMM brain atlas. Original IDs were remapped to be consecutive.

**non-cortical** structure	abbreviation	ID (right/left)
3rd ventricle	3rdVent	1
4th Ventricle	4thVent	2
Accumbens Area	AccA	3/4
Amygdala	Am	5/6
Brain Stem	BS	7
Caudate	Cau	8/9
Cerebellum Exterior	CblmExt	10/11
Cerebellum White Matter	CblmWM	12/13
Cerebral Exterior ^{excluded}	*CrblExt*	*14/15*
Cerebral White Matter	CrblWM	16/17
Cerebrospinal Fluid	CSF	18
Hippocampus	Hc	19/20
Inf. Lateral Ventricle	infLV	21/22
Lateral Ventricle	LV	23/24
Pallidum	Pa	25/26
Putamen	Pu	27/28
Thalamus Proper	Th	29/30
Ventral DC	vDC	31/32
Vessel ^{excluded}	*Vsl*	*33/34*
Optic Chiasm	OptC	35
Cerebellar Vermal Lobules I-V	CVL1t5	36
Cerebellar Vermal Lobules VI-VII	CVL6t7	37
Cerebellar Vermal Lobules VIII-X	CVL8t10	38
Basal Forebrain	BF	40/39

Table 2. Structure names of cortical structures in the NMM brain atlas.

**cortical** structure	abbreviation	ID (right/left)
anterior cingulate gyrus	ACgG	41/42
anterior insula	AIns	43/44
anterior orbital gyrus	AOrG	45/46
angular gyrus	AnG	47/48
calcarine cortex	Calc	49/50
central operculum	CO	51/52
cuneus	Cun	53/54
entorhinal area	EntA	55/56
frontal operculum	FO	57/58
frontal pole	FRP	59/60
fusiform gyrus	FuG	61/62
gyrus rectus	GRe	63/64
inferior occipital gyrus	IOG	65/66
inferior temporal gyrus	ITG	67/68
lingual gyrus	LiG	69/70
lateral orbital gyrus	LOrG	71/72
middle cingulate gyrus	MCgG	73/74
medial frontal cortex	MFC	75/76
middle frontal gyrus	MFG	77/78
middle occipital gyrus	MOG	79/80
medial orbital gyrus	MOrG	81/82
postcentral gyrus medial segment	MPoG	83/84
precentral gyrus medial segment	MPrG	85/86
superior frontal gyrus medial segment	MSFG	87/88
middle temporal gyrus	MTG	89/90
occipital pole	OCP	91/92
occipital fusiform gyrus	OFuG	93/94
opercular part of the inferior frontal gyrus	OpIFG	95/96
orbital part of the inferior frontal gyrus	OrIFG	97/98
posterior cingulate gyrus	PCgG	99/100
precuneus	PCu	101/102
parahippocampal gyrus	PHG	103/104
posterior insula	PIns	105/106
parietal operculum	PO	107/108
postcentral gyrus	PoG	109/110
posterior orbital gyrus	POrG	111/112
planum polare	PP	113/114
precentral gyrus	PrG	115/116
planum temporale	PT	117/118
subcallosal area	SCA	119/120
superior frontal gyrus	SFG	121/122
supplementary motor cortex	SMC	123/124
supramarginal gyrus	SMG	125/126
superior occipital gyrus	SOG	127/128
superior parietal lobule	SPL	129/130
superior temporal gyrus	STG	131/132
temporal pole	TMP	133/134
triangular part of the inferior frontal gyrus	TrIFG	135/136
transverse temporal gyrus	TTG	137/138

Cross-sectional analysis at baseline (AD vs. HC, uncorrected volumes)

Table 3. Classification results in % (6-fold CV, LDA 100 runs, RF/SVM 20 runs) for distinguishing between AD and HC. Significant group differences indicated by + ($p < 0.05$) and ++ ($p < 0.001$). Bonferroni-corrected significance in parentheses. Sorted by effect size.

**AD patients** (N = 322, Positives^P) vs. **Healthy Controls** (N = 404, Negatives^N) **(baseline analysis, uncorrected volumes)**								
structure	ACC (bACC)	SENS	SPEC	mean (SD) [mm^3] ^{P}	mean (SD) [mm^3]^N	effect size (d)	p-value	sig. (corr.)
RandomForest (all features)	85 (85)	81	88					
SVM (all features)	90 (90)	86	93					
Gender (female = 0, male = 1)	52 (52)	55	50	0.5 (0.5)	0.5 (0.5)	0.099	0.18349	o (o)
Age	51 (51)	51	51	75.1 (7.7)	74.9 (5.7)	0.027	0.71340	o (o)
(surrogate structures)								
Ventricles	66 (65)	55	75	63295.6 (28189.0)	46227.4 (20971.9)	0.698	<0.00001	++ (++)
CorticalGreyMatter	61 (61)	62	60	491782.0 (56040.1)	519904.6 (50579.3)	0.530	<0.00001	++ (++)
BrainTissue	56 (56)	57	55	1133344.3 (118326.9)	1163549.9 (110731.1)	0.265	0.00042	++ (+)
DeepGreyMatter	55 (55)	57	54	172879.2 (18868.2)	175625.6 (16551.2)	0.156	0.03725	+ (o)
Brain	54 (55)	56	53	1199158.5 (132290.9)	1212115.6 (118623.9)	0.104	0.16526	o (o)
WhiteMatter	48 (47)	47	48	468683.1 (62373.7)	468019.7 (60538.5)	0.011	0.88497	o (o)
(individual structures)								
LeftHippocampus	76 (76)	76	75	2608.0 (479.7)	3221.8 (434.3)	1.349	<0.00001	++ (++)
Amygdala	75 (75)	73	78	1825.9 (388.6)	2296.6 (314.5)	1.348	<0.00001	++ (++)
Hippocampus	75 (75)	75	75	5429.2 (943.8)	6601.4 (825.8)	1.332	<0.00001	++ (++)
RightAmygdala	75 (75)	73	76	925.6 (209.5)	1168.0 (171.6)	1.280	<0.00001	++ (++)
EntA	73 (73)	71	75	3427.5 (675.5)	4245.4 (609.2)	1.279	<0.00001	++ (++)
LeftAmygdala	75 (75)	73	77	900.3 (205.1)	1128.6 (162.7)	1.250	<0.00001	++ (++)
RightHippocampus	73 (73)	71	75	2821.2 (546.5)	3379.6 (427.4)	1.154	<0.00001	++ (++)
InfLatVent	73 (72)	58	85	3432.1 (1556.6)	2107.1 (825.3)	1.099	<0.00001	++ (++)
LeftInfLatVent	75 (74)	63	85	1652.9 (765.4)	1006.8 (420.7)	1.079	<0.00001	++ (++)
RightInfLatVent	70 (68)	55	82	1779.3 (902.2)	1100.3 (447.5)	0.988	<0.00001	++ (++)
ITG	66 (66)	66	65	21776.6 (3325.8)	24529.3 (2916.2)	0.887	<0.00001	++ (++)
PHG	66 (66)	68	65	6447.4 (977.5)	7217.3 (899.2)	0.824	<0.00001	++ (++)
AnG	67 (67)	67	67	15507.5 (2580.5)	17559.5 (2480.9)	0.812	<0.00001	++ (++)
MTG	64 (64)	66	62	25677.9 (4030.6)	28511.8 (3713.2)	0.735	<0.00001	++ (++)
TMP	64 (63)	61	65	13730.4 (2468.4)	15339.5 (2004.0)	0.724	<0.00001	++ (++)
LateralVentricle	65 (64)	55	73	55184.7 (26190.1)	39883.0 (19481.1)	0.674	<0.00001	++ (++)
LeftLateralVentricle	66 (65)	55	75	29062.5 (14264.5)	20827.4 (10483.3)	0.669	<0.00001	++ (++)
SMG	62 (63)	64	61	13731.4 (2077.6)	15086.0 (2006.8)	0.664	<0.00001	++ (++)
RightLateralVentricle	63 (62)	54	71	26122.3 (12557.2)	19055.6 (9403.3)	0.647	<0.00001	++ (++)
STG	63 (63)	62	63	12671.1 (2031.8)	13889.0 (1933.7)	0.616	<0.00001	++ (++)
OFuG	60 (60)	61	60	7382.1 (1324.3)	8129.7 (1267.7)	0.578	<0.00001	++ (++)
ThalamusProper	61 (61)	63	60	12486.4 (1477.5)	13304.8 (1426.0)	0.565	<0.00001	++ (++)
3rdVentricle	60 (60)	54	65	2319.7 (828.7)	1891.2 (721.5)	0.556	<0.00001	++ (++)
BasalForebrain	62 (62)	64	60	795.6 (215.5)	914.0 (213.2)	0.552	<0.00001	++ (++)
FuG	60 (61)	63	59	15951.4 (2428.3)	17131.2 (2281.3)	0.503	<0.00001	++ (++)
AIns	62 (62)	63	60	8241.7 (1225.1)	8820.0 (1142.4)	0.490	<0.00001	++ (++)
AccumbensArea	60 (60)	57	62	617.0 (184.6)	695.7 (148.3)	0.475	<0.00001	++ (++)
MOG	59 (59)	61	57	10087.5 (1773.4)	10837.5 (1524.1)	0.457	<0.00001	++ (++)
IOG	58 (58)	57	59	11413.2 (2016.2)	12233.2 (1815.4)	0.430	<0.00001	++ (++)
SPL	58 (58)	59	57	16943.2 (2469.3)	17977.7 (2441.6)	0.422	<0.00001	++ (++)
PIns	58 (59)	60	57	4482.8 (701.8)	4771.4 (703.1)	0.411	<0.00001	++ (++)
PP	58 (58)	60	55	3682.5 (589.7)	3912.8 (549.4)	0.406	<0.00001	++ (++)
FO	57 (57)	58	55	3504.1 (549.2)	3718.8 (539.9)	0.395	<0.00001	++ (++)
MFG	59 (59)	60	59	33109.0 (5125.3)	34968.2 (4791.0)	0.376	<0.00001	++ (++)
SFG	56 (57)	58	55	26326.6 (3837.4)	27676.8 (3441.8)	0.373	<0.00001	++ (++)
PT	57 (57)	61	54	3658.7 (712.6)	3926.4 (769.4)	0.359	<0.00001	++ (++)
TrIFG	54 (54)	55	54	6113.5 (1082.3)	6494.0 (1071.5)	0.354	<0.00001	++ (++)
Caudate	58 (56)	43	69	7535.3 (2705.8)	6812.7 (1705.2)	0.328	0.00001	++ (+)
Putamen	56 (56)	57	55	7455.9 (1342.8)	7878.8 (1331.8)	0.316	0.00003	++ (+)
SCA	56 (57)	58	55	2367.0 (496.3)	2510.7 (434.7)	0.310	0.00004	++ (+)
PCu	55 (56)	58	53	21742.5 (3583.1)	22743.6 (3039.0)	0.304	0.00005	++ (+)
PCgG	56 (56)	57	55	8494.1 (1311.2)	8862.3 (1128.5)	0.304	0.00005	++ (+)
CSF	57 (56)	51	61	2419.4 (579.6)	2250.7 (598.6)	0.286	0.00014	++ (+)
POrG	55 (55)	57	54	6068.4 (927.5)	6312.4 (859.2)	0.274	0.00026	++ (+)
MCgG	55 (54)	48	60	10326.7 (1674.5)	9905.0 (1511.3)	0.266	0.00040	++ (+)
MSFG	56 (57)	61	53	12685.2 (2000.5)	13151.5 (1735.4)	0.251	0.00082	++ (o)
SOG	55 (55)	57	54	7063.8 (1242.6)	7354.8 (1136.5)	0.246	0.00106	+ (o)
MFC	54 (54)	54	54	3510.2 (716.9)	3663.4 (603.6)	0.233	0.00185	+ (o)
OCP	53 (53)	57	50	4494.6 (931.2)	4728.0 (1076.0)	0.230	0.00215	+ (o)
CerebellarVermalLobulesI-V	54 (54)	56	52	4464.0 (672.4)	4616.9 (672.9)	0.227	0.00244	+ (o)
OrIFG	55 (55)	60	50	2749.4 (587.8)	2879.5 (568.2)	0.225	0.00263	+ (o)
LOrG	54 (55)	57	52	3713.5 (688.9)	3853.3 (600.1)	0.218	0.00363	+ (o)
OpIFG	52 (53)	57	49	5649.2 (1036.1)	5864.9 (1065.5)	0.205	0.00624	+ (o)
GRe	53 (53)	55	52	3228.0 (549.0)	3335.1 (512.8)	0.202	0.00691	+ (o)
PO	54 (55)	58	52	4251.3 (792.1)	4409.9 (796.3)	0.200	0.00770	+ (o)
CerebellumWhiteMatter	54 (52)	40	65	32795.7 (8328.0)	31344.3 (7506.8)	0.184	0.01393	+ (o)
SMC	53 (53)	57	49	10285.9 (1713.6)	10567.8 (1418.9)	0.181	0.01558	+ (o)
AOrG	51 (51)	52	50	3804.7 (617.0)	3906.9 (598.1)	0.168	0.02448	+ (o)
TTG	56 (57)	62	52	2387.0 (557.6)	2477.1 (532.0)	0.166	0.02684	+ (o)
CerebellarVermalLobulesVI-VII	53 (53)	50	55	2417.3 (432.6)	2355.7 (390.0)	0.150	0.04435	+ (o)
PoG	53 (53)	55	51	18093.5 (2551.3)	18449.6 (2321.8)	0.147	0.04984	+ (o)
MPoG	54 (54)	56	52	1644.7 (394.0)	1699.2 (370.6)	0.143	0.05604	o (o)
PrG	52 (52)	53	51	23676.0 (3462.6)	24096.7 (3052.7)	0.130	0.08268	o (o)
CO	53 (53)	55	51	7785.8 (1114.5)	7923.9 (1090.1)	0.125	0.09374	o (o)
Calc	53 (53)	50	55	7085.9 (1722.2)	6884.2 (1531.2)	0.125	0.09586	o (o)
FRP	51 (52)	53	50	5444.2 (1113.1)	5565.8 (996.7)	0.116	0.12123	o (o)
MPrG	53 (53)	55	51	5428.2 (957.0)	5531.1 (847.0)	0.115	0.12515	o (o)
MOrG	52 (52)	54	51	8243.5 (1150.7)	8361.5 (1010.3)	0.110	0.14236	o (o)
ACg	51 (51)	54	49	8487.7 (1576.8)	8613.8 (1458.5)	0.083	0.26466	o (o)
VentralDC	51 (51)	52	50	8448.8 (965.4)	8523.1 (931.8)	0.079	0.29369	o (o)
BrainStem	50 (50)	52	49	18742.3 (2233.9)	18885.5 (2141.8)	0.066	0.38005	o (o)
Pallidum	51 (51)	49	52	2668.2 (454.5)	2645.4 (394.7)	0.054	0.47061	o (o)
Cun	49 (49)	49	48	10691.9 (2079.7)	10753.0 (1794.8)	0.032	0.67110	o (o)
CerebellarVermalLobulesVIII-X	48 (48)	47	49	2823.6 (474.2)	2814.5 (428.5)	0.020	0.78685	o (o)
4thVentricle	48 (48)	45	51	2359.0 (691.3)	2346.0 (690.8)	0.019	0.80132	o (o)
CerebralWhiteMatter	48 (48)	49	48	435887.4 (58972.0)	436675.4 (56880.4)	0.014	0.85528	o (o)
CerebellumExterior	48 (48)	48	49	97168.8 (12890.5)	97279.6 (11151.4)	0.009	0.90128	o (o)
LiG	48 (48)	47	48	18512.9 (2794.7)	18524.4 (2476.3)	0.004	0.95334	o (o)

Cross-sectional analysis at baseline (AD vs. HC, corrected volumes)

Table 4. Classification results in % (6-fold CV, LDA 100 runs, RF/SVM 20 runs) for distinguishing between AD and HC. Significant group differences indicated by + ($p < 0.05$) and ++ ($p < 0.001$). Bonferroni-corrected significance in parentheses. Sorted by effect size. Mean also shown in % with respect to sample-specific reference volume used for feature correction.

**AD patients** (N = 322, Positives^{P}) vs. **Healthy Controls** (N = 404, Negatives^{N}) **(baseline analysis, †volumes corrected for age/gender/brain size)**								
structure	ACC (bACC)	SENS	SPEC	mean [rel. to HC] (SD) [mm^3]^{P,†}	mean (SD) [mm^3]^{N,†}	effect size (d)	p-value	sig. (corr.)
RandomForest (all features)	87 (86)	83	90					
SVM (all features)	90 (89)	86	92					
Gender (female = 0, male = 1)	52 (52)	55	50	0.5 (0.5)	0.5 (0.5)	0.099	0.18349	o (o)
Age	50 (50)	50	50	75.1 (7.7)	74.9 (5.7)	0.027	0.71340	o (o)
(surrogate structures)								
BrainTissue	72 (71)	63	78	-17942.8 [≙ -1.5%] (22718.7)	0 (17403.6)	0.900	<0.00001	++ (++)
CorticalGreyMatter	68 (67)	63	72	-23635.9 [≙ -4.6%] (28907.3)	0 (24078.1)	0.898	<0.00001	++ (++)
Ventricles	72 (71)	63	79	17757.3 [≙ 46.2%] (22639.6)	0 (17293.9)	0.895	<0.00001	++ (++)
WhiteMatter	57 (56)	51	61	7242.2 [≙ 1.7%] (29309.8)	0 (28686.1)	0.250	0.00086	++ (o)
DeepGreyMatter	52 (52)	50	54	-1549.1 [≙ -0.9%] (13310.2)	0 (11142.2)	0.127	0.08834	o (o)
Brain	54 (55)	56	53	1199158.5 (132290.9)	1212115.6 (118623.9)	0.104	0.16526	o (o)
(individual structures)								
Amygdala	80 (80)	76	84	-452.0 [≙ -20.0%] (332.0)	0 (250.5)	1.561	<0.00001	++ (++)
Hippocampus	78 (78)	75	80	-1115.4 [≙ -17.0%] (817.7)	0 (660.7)	1.519	<0.00001	++ (++)
EntA	78 (78)	76	80	-801.3 [≙ -19.0%] (583.3)	0 (485.3)	1.509	<0.00001	++ (++)
LeftHippocampus	79 (78)	76	81	-588.1 [≙ -18.4%] (423.1)	0 (364.6)	1.502	<0.00001	++ (++)
RightAmygdala	80 (80)	77	83	-232.2 [≙ -20.2%] (182.6)	0 (139.4)	1.452	<0.00001	++ (++)
LeftAmygdala	78 (78)	75	81	-219.8 [≙ -19.7%] (179.7)	0 (135.5)	1.403	<0.00001	++ (++)
RightHippocampus	76 (75)	71	79	-527.3 [≙ -15.7%] (488.0)	0 (339.1)	1.280	<0.00001	++ (++)
InfLatVent	78 (77)	65	89	1330.8 [≙ 65.8%] (1367.4)	0 (702.2)	1.267	<0.00001	++ (++)
LeftInfLatVent	77 (76)	66	86	649.5 [≙ 68.2%] (677.1)	0 (360.6)	1.237	<0.00001	++ (++)
ITG	71 (71)	69	73	-2588.0 [≙ -10.7%] (2506.9)	0 (1954.5)	1.168	<0.00001	++ (++)
RightInfLatVent	75 (73)	59	87	681.3 [≙ 63.6%] (814.9)	0 (391.3)	1.106	<0.00001	++ (++)
PHG	68 (68)	64	72	-739.8 [≙ -10.3%] (832.6)	0 (702.4)	0.970	<0.00001	++ (++)
MTG	69 (69)	69	70	-2534.0 [≙ -9.0%] (2853.0)	0 (2411.3)	0.969	<0.00001	++ (++)
AnG	68 (67)	65	70	-1880.5 [≙ -10.6%] (2345.6)	0 (1973.2)	0.876	<0.00001	++ (++)
TMP	66 (65)	64	67	-1521.7 [≙ -10.1%] (1967.2)	0 (1539.3)	0.873	<0.00001	++ (++)
LateralVentricle	71 (70)	62	78	15972.2 [≙ 50.5%] (21196.2)	0 (16214.4)	0.859	<0.00001	++ (++)
LeftLateralVentricle	70 (69)	61	76	8584.7 [≙ 51.4%] (11586.9)	0 (8816.1)	0.847	<0.00001	++ (++)
RightLateralVentricle	70 (69)	59	78	7387.5 [≙ 49.6%] (10387.1)	0 (7883.2)	0.814	<0.00001	++ (++)
STG	65 (65)	64	66	-1100.3 [≙ -7.9%] (1610.7)	0 (1478.6)	0.715	<0.00001	++ (++)
3rdVentricle	67 (66)	63	70	434.3 [≙ 27.9%] (699.9)	0 (591.1)	0.677	<0.00001	++ (++)
SMG	64 (64)	63	65	-1196.5 [≙ -7.8%] (2003.1)	0 (1697.8)	0.650	<0.00001	++ (++)
ThalamusProper	63 (63)	61	65	-680.6 [≙ -5.0%] (1214.1)	0 (1005.2)	0.617	<0.00001	++ (++)
FuG	62 (62)	62	61	-1072.1 [≙ -6.4%] (1750.6)	0 (1769.3)	0.609	<0.00001	++ (++)
OFuG	60 (60)	58	62	-690.7 [≙ -8.4%] (1225.8)	0 (1061.2)	0.607	<0.00001	++ (++)
BasalForebrain	63 (63)	66	61	-109.6 [≙ -12.0%] (195.4)	0 (189.3)	0.571	<0.00001	++ (++)
AIns	59 (60)	62	58	-509.3 [≙ -5.8%] (995.9)	0 (915.4)	0.535	<0.00001	++ (++)
MOG	60 (60)	61	59	-643.3 [≙ -6.1%] (1375.1)	0 (1195.7)	0.503	<0.00001	++ (++)
IOG	59 (59)	60	59	-697.2 [≙ -5.8%] (1571.8)	0 (1386.2)	0.474	<0.00001	++ (++)
AccumbensArea	60 (60)	59	61	-71.7 [≙ -10.3%] (177.2)	0 (138.5)	0.457	<0.00001	++ (++)
PP	58 (58)	61	56	-200.0 [≙ -5.2%] (464.5)	0 (433.4)	0.447	<0.00001	++ (++)
MCgG	58 (57)	54	60	544.3 [≙ 5.6%] (1270.6)	0 (1190.1)	0.444	<0.00001	++ (++)
PIns	58 (58)	58	58	-231.1 [≙ -4.9%] (524.6)	0 (547.9)	0.430	<0.00001	++ (++)
MFG	58 (58)	56	60	-1467.9 [≙ -4.2%] (3689.2)	0 (3194.1)	0.429	<0.00001	++ (++)
SPL	56 (56)	57	56	-867.5 [≙ -4.7%] (2175.8)	0 (1972.5)	0.420	<0.00001	++ (++)
SFG	59 (59)	60	58	-1124.3 [≙ -4.1%] (3032.2)	0 (2632.7)	0.399	<0.00001	++ (++)
Caudate	58 (57)	46	68	799.5 [≙ 11.2%] (2447.8)	0 (1597.5)	0.396	<0.00001	++ (++)
FO	60 (60)	59	60	-187.3 [≙ -4.9%] (506.5)	0 (458.7)	0.390	<0.00001	++ (++)
PT	56 (56)	58	55	-232.4 [≙ -5.8%] (590.0)	0 (636.4)	0.377	<0.00001	++ (++)
TrIFG	57 (57)	56	58	-331.8 [≙ -5.0%] (985.1)	0 (933.4)	0.347	<0.00001	++ (++)
PCu	56 (56)	57	54	-788.1 [≙ -3.6%] (2502.1)	0 (2124.3)	0.343	<0.00001	++ (++)
PCgG	57 (57)	59	56	-306.1 [≙ -3.7%] (960.5)	0 (898.4)	0.330	0.00001	++ (++)
SCA	58 (58)	58	59	-124.5 [≙ -5.1%] (441.7)	0 (388.6)	0.301	0.00006	++ (+)
POrG	57 (57)	54	59	-211.0 [≙ -3.4%] (738.1)	0 (685.9)	0.297	0.00008	++ (+)
CSF	58 (58)	53	62	172.5 [≙ 9.3%] (621.2)	0 (545.8)	0.297	0.00008	++ (+)
Putamen	57 (57)	58	56	-367.5 [≙ -4.6%] (1300.2)	0 (1243.1)	0.290	0.00012	++ (+)
MSFG	54 (54)	55	53	-349.9 [≙ -2.8%] (1447.4)	0 (1334.5)	0.253	0.00076	++ (o)
MFC	53 (53)	54	53	-123.9 [≙ -3.6%] (577.8)	0 (493.6)	0.233	0.00192	+ (o)
CerebellumWhiteMatter	55 (54)	44	64	1677.9 [≙ 5.8%] (7982.2)	0 (6947.8)	0.226	0.00257	+ (o)
OCP	53 (54)	57	50	-207.2 [≙ -4.5%] (815.9)	0 (999.5)	0.225	0.00274	+ (o)
SOG	55 (56)	58	53	-220.0 [≙ -3.1%] (1044.7)	0 (974.8)	0.219	0.00354	+ (o)
CerebralWhiteMatter	56 (55)	52	58	5564.3 [≙ 1.4%] (26392.1)	0 (25736.3)	0.214	0.00434	+ (o)
OrIFG	55 (56)	59	52	-109.5 [≙ -3.9%] (518.0)	0 (510.3)	0.213	0.00446	+ (o)
LOrG	52 (52)	53	51	-111.8 [≙ -3.0%] (565.6)	0 (492.2)	0.213	0.00456	+ (o)
CerebellarVermalLobulesI-V	56 (56)	57	55	-128.8 [≙ -2.8%] (620.1)	0 (632.2)	0.205	0.00610	+ (o)
Calc	53 (53)	51	55	275.0 [≙ 3.8%] (1536.7)	0 (1432.9)	0.186	0.01309	+ (o)
CerebellarVermalLobulesVI-VII	54 (54)	52	55	71.0 [≙ 3.0%] (401.3)	0 (370.4)	0.185	0.01368	+ (o)
OpIFG	51 (51)	53	50	-153.5 [≙ -2.5%] (890.2)	0 (909.9)	0.170	0.02290	+ (o)
GRe	52 (52)	54	51	-77.1 [≙ -2.5%] (463.7)	0 (463.0)	0.166	0.02614	+ (o)
SMC	52 (52)	54	51	-192.1 [≙ -2.0%] (1309.9)	0 (1108.0)	0.160	0.03273	+ (o)
PO	53 (53)	54	52	-105.0 [≙ -2.1%] (682.3)	0 (637.7)	0.160	0.03303	+ (o)
AOrG	52 (52)	52	52	-71.6 [≙ -1.8%] (483.0)	0 (464.7)	0.151	0.04301	+ (o)
TTG	54 (54)	56	53	-70.4 [≙ -2.7%] (481.9)	0 (455.3)	0.151	0.04398	+ (o)
MPoG	53 (53)	53	53	-44.8 [≙ -2.5%] (377.8)	0 (348.2)	0.124	0.09769	o (o)
PrG	52 (53)	55	50	-262.1 [≙ -1.1%] (2723.4)	0 (2472.1)	0.101	0.17534	o (o)
Pallidum	54 (54)	55	53	38.0 [≙ 1.4%] (418.7)	0 (362.0)	0.098	0.19090	o (o)
PoG	52 (52)	52	52	-189.2 [≙ -1.0%] (2124.4)	0 (1913.6)	0.094	0.20803	o (o)
FRP	50 (50)	53	47	-76.0 [≙ -1.5%] (962.3)	0 (901.5)	0.082	0.27406	o (o)
MPrG	51 (51)	53	50	-58.2 [≙ -1.1%] (819.9)	0 (747.4)	0.075	0.31847	o (o)
CO	50 (50)	51	49	-58.3 [≙ -0.7%] (783.8)	0 (796.8)	0.074	0.32384	o (o)
MOrG	51 (51)	53	49	-61.3 [≙ -0.7%] (943.0)	0 (819.7)	0.070	0.34945	o (o)
CerebellarVermalLobulesVIII-X	51 (51)	51	51	22.1 [≙ 0.8%] (423.1)	0 (375.1)	0.056	0.45705	o (o)
LiG	51 (51)	51	51	106.4 [≙ 0.5%] (2022.0)	0 (1846.7)	0.055	0.46010	o (o)
CerebellumExterior	52 (52)	52	51	442.1 [≙ 0.4%] (10408.6)	0 (8931.0)	0.046	0.53842	o (o)
4thVentricle	50 (50)	46	54	19.9 [≙ 1.1%] (646.4)	0 (643.3)	0.031	0.67923	o (o)
Cun	48 (48)	46	49	33.2 [≙ 0.2%] (1619.0)	0 (1403.3)	0.022	0.76765	o (o)
VentralDC	47 (47)	46	47	-13.7 [≙ -0.0%] (709.3)	0 (597.0)	0.021	0.77728	o (o)
BrainStem	48 (48)	47	48	17.8 [≙ 0.2%] (1574.8)	0 (1484.5)	0.012	0.87624	o (o)
ACg	47 (47)	47	48	-5.9 [≙ -0.0%] (1178.5)	0 (1101.8)	0.005	0.94417	o (o)

Cross-sectional analysis at baseline (pMCI vs. sMCI, corrected volumes)

Table 5. Classification results in % (6-fold CV, LDA 100 runs, RF/SVM 20 runs) for distinguishing between pMCI and sMCI. Significant group differences indicated by + ($p < 0.05$) and ++ ($p < 0.001$). Bonferroni-corrected significance in parentheses. Sorted by effect size. Mean also shown in % with respect to sample-specific reference volume used for feature correction.

**progressive MCI** (N = 177, Positives^{P}) vs. **stable MCI** (N = 166, Negatives^{N}) **(baseline analysis, †volumes corrected for age/gender/brain size)**								
structure	ACC (bACC)	SENS	SPEC	mean [rel. to HC] (SD) $[mm^3]^{P,\dagger}$	mean [rel. to HC] (SD) $[mm^3]^{N,\dagger}$	effect size (d)	p-value	sig. (corr.)
RandomForest (all features)	68 (68)	72	64					
SVM (all features)	67 (67)	70	64					
Age	47 (47)	45	48	73.5 (7.6)	73.8 (7.6)	0.029	0.78516	o (o)
Gender (female = 0, male = 1)	45 (45)	45	45	0.6 (0.5)	0.6 (0.5)	0.006	0.95829	o (o)
(surrogate structures)								
BrainTissue	60 (60)	51	69	-12652.2 [≙ -1.1%] (20144.6)	-4372.1 [≙ -0.4%] (20741.3)	0.405	0.00021	++ (+)
Ventricles	60 (60)	51	70	12483.5 [≙ 35.6%] (20045.3)	4427.7 [≙ 9.7%] (20676.8)	0.396	0.00029	++ (+)
CorticalGreyMatter	56 (56)	55	56	-16743.9 [≙ -3.2%] (27348.8)	-6929.4 [≙ -1.3%] (26948.6)	0.361	0.00091	++ (o)
Brain	51 (51)	52	49	1222424.9 (131084.2)	1238635.5 (118830.2)	0.129	0.23200	o (o)
WhiteMatter	48 (48)	47	49	5966.3 [≙ 1.3%] (27919.1)	4607.3 [≙ 1.0%] (26735.5)	0.050	0.64592	o (o)
DeepGreyMatter	47 (47)	46	48	-1874.6 [≙ -1.0%] (11838.0)	-2050.0 [≙ -1.1%] (11988.8)	0.015	0.89167	o (o)
(individual structures)								
Amygdala	65 (65)	63	68	-387.4 [≙ -16.5%] (308.7)	-149.4 [≙ -6.4%] (352.2)	0.720	<0.00001	++ (++)
LeftAmygdala	62 (62)	61	64	-191.6 [≙ -16.7%] (161.2)	-70.4 [≙ -6.0%] (189.7)	0.690	<0.00001	++ (++)
InfLatVent	64 (64)	54	74	1034.0 [≙ 53.8%] (1097.9)	358.6 [≙ 17.4%] (903.1)	0.670	<0.00001	++ (++)
LeftInfLatVent	65 (65)	56	75	480.0 [≙ 53.9%] (538.2)	156.3 [≙ 15.8%] (451.1)	0.650	<0.00001	++ (++)
RightAmygdala	65 (65)	63	67	-195.8 [≙ -16.4%] (176.7)	-79.0 [≙ -6.7%] (184.1)	0.648	<0.00001	++ (++)
EntA	61 (61)	61	60	-678.3 [≙ -15.6%] (565.7)	-294.0 [≙ -6.7%] (622.8)	0.647	<0.00001	++ (++)
RightInfLatVent	64 (64)	54	74	554.0 [≙ 53.9%] (689.1)	202.3 [≙ 18.9%] (527.5)	0.571	<0.00001	++ (++)
Hippocampus	61 (61)	63	60	-1042.8 [≙ -15.6%] (817.1)	-604.7 [≙ -8.9%] (786.1)	0.546	<0.00001	++ (++)
RightHippocampus	63 (63)	61	64	-514.7 [≙ -15.1%] (442.9)	-287.7 [≙ -8.3%] (408.3)	0.532	<0.00001	++ (++)
MTG	60 (60)	59	60	-2305.3 [≙ -7.9%] (2698.1)	-925.8 [≙ -3.0%] (2708.9)	0.510	<0.00001	++ (++)
AnG	60 (60)	61	59	-1579.0 [≙ -8.9%] (2223.0)	-495.7 [≙ -2.6%] (2084.0)	0.502	<0.00001	++ (++)
LeftHippocampus	61 (61)	62	60	-528.1 [≙ -16.2%] (429.6)	-317.0 [≙ -9.6%] (425.8)	0.493	<0.00001	++ (++)
CSF	59 (59)	54	64	156.4 [≙ 7.6%] (533.9)	-61.6 [≙ -2.2%] (491.3)	0.424	0.00010	++ (+)
3rdVentricle	59 (59)	55	64	376.2 [≙ 24.3%] (661.3)	113.9 [≙ 6.1%] (639.9)	0.403	0.00023	++ (+)
LateralVentricle	60 (61)	51	70	11080.2 [≙ 40.1%] (18700.0)	3986.3 [≙ 10.1%] (19536.4)	0.371	0.00066	++ (o)
RightLateralVentricle	60 (60)	51	69	5078.1 [≙ 38.4%] (9151.1)	1656.3 [≙ 9.1%] (9510.1)	0.367	0.00077	++ (o)
LeftLateralVentricle	59 (59)	51	68	6002.1 [≙ 41.6%] (10336.4)	2330.0 [≙ 11.1%] (10722.7)	0.349	0.00136	+ (o)
STG	57 (57)	57	56	-1048.8 [≙ -7.4%] (1509.9)	-513.8 [≙ -3.4%] (1583.2)	0.346	0.00149	+ (o)
ITG	57 (57)	57	56	-1510.5 [≙ -6.1%] (2300.7)	-691.2 [≙ -2.8%] (2482.3)	0.343	0.00165	+ (o)
TMP	58 (58)	58	58	-875.3 [≙ -5.7%] (1703.2)	-310.1 [≙ -1.9%] (1766.7)	0.326	0.00275	+ (o)
MCgG	55 (55)	50	60	472.7 [≙ 4.6%] (1159.1)	78.4 [≙ 0.7%] (1292.7)	0.322	0.00311	+ (o)
BasalForebrain	57 (57)	57	57	-108.9 [≙ -11.4%] (190.9)	-45.6 [≙ -4.7%] (207.0)	0.318	0.00346	+ (o)
IOG	56 (56)	59	52	-466.0 [≙ -3.9%] (1583.2)	17.9 [≙ -0.0%] (1546.6)	0.309	0.00449	+ (o)
FuG	56 (56)	57	55	-776.1 [≙ -4.5%] (1755.3)	-257.5 [≙ -1.5%] (1632.4)	0.306	0.00495	+ (o)
MOG	58 (58)	59	56	-445.8 [≙ -4.3%] (1531.4)	-25.6 [≙ -0.3%] (1245.4)	0.300	0.00579	+ (o)
OFuG	56 (56)	55	57	-415.3 [≙ -4.9%] (1153.0)	-57.9 [≙ -0.6%] (1242.7)	0.299	0.00604	+ (o)
AccumbensArea	59 (59)	55	63	-66.5 [≙ -9.6%] (162.8)	-24.3 [≙ -3.4%] (137.4)	0.279	0.01012	+ (o)
ThalamusProper	56 (55)	57	54	-650.7 [≙ -4.8%] (1342.2)	-310.3 [≙ -2.3%] (1187.7)	0.268	0.01356	+ (o)
Putamen	59 (59)	59	60	-356.0 [≙ -4.5%] (1372.3)	-28.0 [≙ -0.4%] (1071.7)	0.265	0.01454	+ (o)
SMG	53 (53)	53	53	-880.2 [≙ -5.8%] (1933.5)	-376.0 [≙ -2.4%] (1924.4)	0.261	0.01608	+ (o)
OrIFG	58 (58)	60	55	-111.1 [≙ -3.6%] (518.9)	16.4 [≙ 0.6%] (489.0)	0.253	0.01998	+ (o)
PIns	58 (58)	59	57	-216.3 [≙ -4.6%] (569.6)	-82.3 [≙ -1.6%] (486.6)	0.252	0.02010	+ (o)
AIns	56 (55)	60	51	-403.2 [≙ -4.5%] (892.4)	-175.9 [≙ -1.9%] (937.7)	0.248	0.02206	+ (o)
FO	58 (58)	61	54	-189.9 [≙ -5.0%] (473.2)	-73.5 [≙ -1.9%] (468.1)	0.247	0.02266	+ (o)
FRP	55 (55)	55	55	-178.4 [≙ -3.2%] (995.6)	58.1 [≙ 0.9%] (978.8)	0.239	0.02731	+ (o)
PCgG	53 (53)	57	50	-176.8 [≙ -2.1%] (851.6)	35.2 [≙ 0.3%] (947.5)	0.236	0.02984	+ (o)
SFG	51 (51)	50	53	-891.9 [≙ -3.1%] (2741.4)	-274.6 [≙ -0.9%] (2842.4)	0.221	0.04139	+ (o)
MSFG	55 (55)	54	56	-383.6 [≙ -3.0%] (1538.4)	-69.4 [≙ -0.6%] (1404.0)	0.213	0.04948	+ (o)
PO	58 (58)	58	57	-150.2 [≙ -3.2%] (668.2)	-12.6 [≙ -0.2%] (636.3)	0.211	0.05185	o (o)
PrG	53 (53)	51	54	327.2 [≙ 1.2%] (3003.8)	-269.9 [≙ -1.1%] (2644.4)	0.211	0.05213	o (o)
SPL	55 (55)	57	53	-513.7 [≙ -2.8%] (2105.3)	-91.4 [≙ -0.4%] (1985.5)	0.206	0.05715	o (o)
PCu	55 (55)	54	57	-543.4 [≙ -2.4%] (2447.9)	-80.3 [≙ -0.4%] (2204.2)	0.198	0.06707	o (o)
PHG	56 (56)	56	55	-553.9 [≙ -7.5%] (799.3)	-403.9 [≙ -5.4%] (766.7)	0.191	0.07742	o (o)
Calc	53 (54)	48	59	313.9 [≙ 4.5%] (1471.6)	72.0 [≙ 0.7%] (1551.3)	0.160	0.13935	o (o)
MFG	49 (49)	51	47	-1109.3 [≙ -3.1%] (3500.4)	-578.0 [≙ -1.6%] (3119.2)	0.160	0.13961	o (o)
SMC	53 (53)	52	53	-71.7 [≙ -0.8%] (1254.1)	-267.8 [≙ -2.4%] (1219.8)	0.158	0.14334	o (o)
Caudate	51 (51)	39	63	490.3 [≙ 7.0%] (2016.4)	172.7 [≙ 2.3%] (2103.6)	0.154	0.15432	o (o)
CerebellarVermalLobulesVI-VII	53 (54)	49	58	62.9 [≙ 2.7%] (350.8)	6.4 [≙ 0.3%] (396.3)	0.151	0.16198	o (o)
BrainStem	50 (51)	49	52	35.3 [≙ 0.2%] (1656.3)	-201.8 [≙ -1.1%] (1493.6)	0.150	0.16584	o (o)
TrIFG	53 (53)	54	51	-328.1 [≙ -4.9%] (972.6)	-183.2 [≙ -2.8%] (958.8)	0.150	0.16586	o (o)
GRe	54 (54)	57	51	-40.2 [≙ -1.3%] (484.7)	31.6 [≙ 0.9%] (507.6)	0.145	0.18101	o (o)
PP	54 (54)	56	53	-209.2 [≙ -5.2%] (444.7)	-153.5 [≙ -3.7%] (405.8)	0.131	0.22693	o (o)
MPrG	49 (49)	47	50	141.9 [≙ 2.4%] (981.1)	32.8 [≙ 0.4%] (760.2)	0.124	0.25265	o (o)
VentralDC	53 (53)	52	53	-55.7 [≙ -0.6%] (672.9)	-132.9 [≙ -1.5%] (624.4)	0.119	0.27208	o (o)
OCP	52 (52)	53	51	25.5 [≙ 0.3%] (1055.1)	136.3 [≙ 2.6%] (908.3)	0.112	0.29959	o (o)
SCA	50 (50)	51	49	-90.4 [≙ -3.6%] (407.2)	-46.3 [≙ -1.9%] (381.1)	0.112	0.30165	o (o)
PT	55 (55)	55	54	-274.9 [≙ -6.7%] (635.1)	-208.9 [≙ -4.9%] (575.4)	0.109	0.31490	o (o)
ACg	51 (51)	52	50	-161.8 [≙ -1.9%] (1157.0)	-40.5 [≙ -0.4%] (1133.5)	0.106	0.32780	o (o)
CerebellumExterior	53 (53)	50	56	271.9 [≙ 0.3%] (9093.7)	-695.3 [≙ -0.7%] (9828.3)	0.102	0.34449	o (o)
OpIFG	52 (52)	50	54	-164.8 [≙ -2.5%] (946.5)	-74.3 [≙ -0.9%] (994.5)	0.093	0.38867	o (o)
LOrG	49 (49)	48	49	-94.9 [≙ -2.2%] (526.9)	-46.4 [≙ -1.0%] (610.2)	0.085	0.43046	o (o)
SOG	52 (51)	54	49	-30.6 [≙ -0.4%] (1105.4)	51.4 [≙ 0.6%] (890.3)	0.081	0.45156	o (o)
MPoG	50 (50)	49	51	-50.0 [≙ -2.9%] (374.9)	-74.2 [≙ -4.1%] (365.0)	0.066	0.54466	o (o)
POrG	51 (51)	54	49	-127.2 [≙ -2.0%] (678.0)	-85.1 [≙ -1.3%] (691.7)	0.061	0.56966	o (o)
PoG	52 (52)	52	52	225.5 [≙ 1.1%] (2211.5)	100.1 [≙ 0.5%] (1992.6)	0.059	0.58233	o (o)
CerebellarVermalLobulesI-V	49 (49)	50	48	-88.1 [≙ -2.0%] (588.0)	-56.4 [≙ -1.3%] (617.9)	0.053	0.62643	o (o)
CerebellumWhiteMatter	49 (49)	42	56	1633.9 [≙ 5.2%] (8349.6)	1274.0 [≙ 3.8%] (6934.3)	0.047	0.66544	o (o)
LiG	49 (49)	49	49	26.2 [≙ 0.2%] (2037.7)	-61.0 [≙ -0.2%] (2098.3)	0.042	0.69632	o (o)
CerebralWhiteMatter	46 (46)	47	46	4332.4 [≙ 1.0%] (25782.2)	3333.3 [≙ 0.8%] (25445.0)	0.039	0.71838	o (o)
4thVentricle	48 (48)	45	52	-6.9 [≙ 0.3%] (571.0)	-31.1 [≙ -0.9%] (681.2)	0.039	0.72152	o (o)
TTG	46 (46)	47	45	-48.6 [≙ -1.7%] (506.4)	-36.1 [≙ -1.3%] (443.2)	0.026	0.80827	o (o)
MFC	48 (48)	49	46	-145.6 [≙ -3.9%] (598.9)	-131.3 [≙ -3.6%] (575.9)	0.024	0.82244	o (o)
Pallidum	47 (47)	48	47	9.3 [≙ 0.2%] (383.8)	2.4 [≙ 0.0%] (364.6)	0.018	0.86516	o (o)
Cun	47 (47)	46	48	129.0 [≙ 1.2%] (1484.4)	102.5 [≙ 1.0%] (1449.4)	0.018	0.86737	o (o)
MOrG	46 (46)	47	45	4.1 [≙ -0.0%] (835.1)	-8.5 [≙ -0.1%] (900.9)	0.015	0.89268	o (o)
CO	46 (46)	46	46	-96.2 [≙ -1.2%] (783.1)	-107.0 [≙ -1.3%] (830.1)	0.013	0.90184	o (o)
CerebellarVermalLobulesVIII-X	46 (46)	47	45	11.7 [≙ 0.5%] (406.8)	17.1 [≙ 0.6%] (421.7)	0.013	0.90479	o (o)
AOrG	46 (46)	46	46	-73.3 [≙ -1.9%] (451.4)	-78.7 [≙ -1.9%] (502.9)	0.011	0.91793	o (o)

Longitudinal analysis (baseline→m12, AD vs. HC)

Table 6. Classification results in % (6-fold CV, LDA 100 runs, RF/SVM 20 runs) for distinguishing between AD and HC. Significant group differences indicated by + ($p < 0.05$) and ++ ($p < 0.001$). Bonferroni-corrected significance in parentheses. Sorted by effect size.

**AD patients** (N = 195, Positives^{P}) vs. **Healthy Controls** (N = 290, Negatives^{N}) **(longitudinal analysis, bl→m12)**								
structure	ACC (bACC)	SENS	SPEC	mean (SD) [%]^{P}	mean (SD) [%]^{N}	effect size (d)	p-value	sig. (corr.)
RandomForest (all features)	85 (84)	78	90					
SVM (all features)	84 (82)	71	93					
Age	54 (54)	53	54	75.3 (7.7)	74.8 (5.6)	0.072	0.43678	o (o)
Gender (female = 0, male = 1)	48 (48)	50	46	0.5 (0.5)	0.5 (0.5)	0.042	0.64808	o (o)
(surrogate structures)								
Ventricles	75 (74)	64	83	7.2 (4.6)	2.8 (3.0)	1.202	<0.00001	++ (++)
BrainTissue	70 (70)	67	72	-1.3 (1.1)	-0.5 (0.8)	0.862	<0.00001	++ (++)
WhiteMatter	73 (73)	68	77	-0.8 (1.0)	-0.2 (0.7)	0.736	<0.00001	++ (++)
CorticalGreyMatter	65 (64)	62	67	-1.9 (2.3)	-0.8 (1.4)	0.585	<0.00001	++ (++)
Brain	64 (64)	63	65	-0.9 (1.0)	-0.4 (0.7)	0.574	<0.00001	++ (++)
DeepGreyMatter	66 (64)	57	71	-1.4 (1.7)	-0.8 (1.3)	0.455	<0.00001	++ (++)
(individual structures)								
Hippocampus	80 (78)	67	88	-4.8 (3.7)	-1.1 (1.7)	1.400	<0.00001	++ (++)
InfLatVent	79 (77)	69	86	7.5 (5.5)	1.8 (3.3)	1.334	<0.00001	++ (++)
LeftHippocampus	80 (78)	70	87	-4.9 (4.2)	-1.1 (1.7)	1.287	<0.00001	++ (++)
MTG	76 (74)	67	82	-3.8 (2.8)	-1.1 (1.5)	1.274	<0.00001	++ (++)
RightInfLatVent	77 (75)	65	84	7.4 (6.4)	1.8 (3.3)	1.185	<0.00001	++ (++)
RightLateralVentricle	75 (73)	63	83	7.5 (5.1)	2.8 (3.1)	1.170	<0.00001	++ (++)
LeftInfLatVent	78 (76)	70	83	7.5 (5.8)	1.7 (4.4)	1.166	<0.00001	++ (++)
LateralVentricle	74 (72)	62	83	7.6 (4.9)	3.0 (3.2)	1.160	<0.00001	++ (++)
RightHippocampus	78 (75)	62	89	-4.8 (4.5)	-1.1 (2.1)	1.138	<0.00001	++ (++)
LeftLateralVentricle	74 (72)	63	81	7.6 (4.9)	3.1 (3.4)	1.085	<0.00001	++ (++)
PP	77 (76)	70	81	-6.7 (5.5)	-2.0 (3.5)	1.075	<0.00001	++ (++)
CerebralWhiteMatter	76 (74)	67	82	-0.9 (0.9)	-0.2 (0.6)	0.914	<0.00001	++ (++)
PT	70 (69)	62	76	-5.8 (5.3)	-2.1 (3.1)	0.898	<0.00001	++ (++)
ITG	72 (71)	67	75	-2.7 (2.7)	-1.0 (1.3)	0.866	<0.00001	++ (++)
FuG	69 (68)	63	73	-1.7 (1.7)	-0.5 (1.1)	0.852	<0.00001	++ (++)
Amygdala	76 (74)	66	83	-3.3 (5.0)	-0.5 (2.0)	0.787	<0.00001	++ (++)
EntA	69 (69)	65	72	-3.7 (4.1)	-1.2 (2.8)	0.747	<0.00001	++ (++)
TMP	72 (71)	67	74	-4.3 (4.6)	-1.8 (2.8)	0.700	<0.00001	++ (++)
LeftAmygdala	73 (70)	58	82	-3.6 (6.2)	-0.5 (2.5)	0.699	<0.00001	++ (++)
SMG	64 (64)	61	67	-2.9 (3.5)	-1.1 (2.1)	0.674	<0.00001	++ (++)
RightAmygdala	74 (73)	67	79	-3.3 (6.1)	-0.5 (2.5)	0.631	<0.00001	++ (++)
TTG	67 (66)	62	70	-4.6 (5.3)	-1.9 (3.6)	0.604	<0.00001	++ (++)
AnG	63 (62)	58	66	-2.6 (3.7)	-0.9 (2.2)	0.598	<0.00001	++ (++)
AIns	65 (64)	59	68	-2.0 (2.7)	-0.7 (1.9)	0.562	<0.00001	++ (++)
MSFG	60 (59)	57	61	-2.4 (3.4)	-0.7 (2.6)	0.551	<0.00001	++ (++)
PHG	61 (60)	57	63	-1.7 (2.5)	-0.6 (1.8)	0.521	<0.00001	++ (++)
Caudate	64 (63)	60	67	-4.7 (5.6)	-2.3 (3.7)	0.512	<0.00001	++ (++)
IOG	64 (63)	58	69	-2.2 (3.4)	-0.9 (2.2)	0.442	<0.00001	++ (++)
SMC	60 (60)	58	62	-1.9 (3.2)	-0.7 (2.5)	0.434	<0.00001	++ (++)
3rdVentricle	63 (62)	57	67	4.1 (4.4)	2.4 (3.6)	0.427	<0.00001	++ (++)
OrIFG	60 (60)	59	62	-2.8 (3.7)	-1.3 (3.6)	0.425	<0.00001	++ (++)
FO	58 (58)	54	61	-1.9 (3.8)	-0.6 (2.3)	0.406	0.00001	++ (+)
PCu	60 (60)	59	60	-1.2 (3.1)	-0.2 (2.0)	0.395	0.00002	++ (+)
PCgG	57 (56)	53	59	-0.8 (1.9)	-0.1 (1.7)	0.385	0.00004	++ (+)
MFG	60 (60)	61	59	-2.0 (3.8)	-0.9 (2.1)	0.384	0.00004	++ (+)
ACg	60 (60)	58	62	-1.6 (3.4)	-0.5 (2.3)	0.384	0.00004	++ (+)
SFG	59 (59)	57	61	-2.1 (4.1)	-0.9 (2.4)	0.354	0.00015	++ (+)
MOrG	59 (58)	54	62	-1.9 (4.5)	-0.6 (3.0)	0.352	0.00016	++ (+)
TrIFG	60 (59)	56	62	-2.4 (3.6)	-1.4 (2.5)	0.348	0.00019	++ (+)
ThalamusProper	72 (71)	63	79	-2.2 (2.3)	-1.1 (3.9)	0.331	0.00038	++ (+)
BrainStem	55 (53)	45	61	-0.9 (1.3)	-0.6 (0.9)	0.310	0.00089	++ (o)
MFC	60 (60)	57	63	-1.8 (4.5)	-0.6 (3.4)	0.303	0.00116	+ (o)
MOG	58 (58)	56	60	-1.7 (4.1)	-0.7 (2.6)	0.299	0.00133	+ (o)
4thVentricle	58 (57)	51	62	2.4 (6.2)	0.9 (4.5)	0.295	0.00152	+ (o)
CSF	56 (55)	54	57	1.1 (3.7)	0.1 (3.2)	0.289	0.00192	+ (o)
OFuG	55 (55)	53	56	-1.0 (3.3)	-0.2 (2.5)	0.277	0.00294	+ (o)
SPL	60 (59)	58	60	-2.0 (4.0)	-1.0 (3.0)	0.274	0.00320	+ (o)
OpIFG	56 (56)	54	58	-2.1 (4.4)	-1.2 (2.8)	0.270	0.00369	+ (o)
POrG	56 (56)	56	56	-1.3 (3.3)	-0.6 (2.4)	0.236	0.01130	+ (o)
CerebellumExterior	60 (58)	49	68	-1.3 (2.6)	-0.8 (2.1)	0.229	0.01382	+ (o)
MPrG	55 (54)	53	56	-1.0 (3.9)	-0.3 (3.2)	0.194	0.03708	+ (o)
LOrG	56 (56)	55	57	-2.7 (4.8)	-1.7 (5.1)	0.192	0.03847	+ (o)
SOG	58 (57)	56	59	-1.6 (4.6)	-0.8 (3.5)	0.187	0.04441	+ (o)
SCA	58 (58)	59	57	-2.6 (10.0)	-1.3 (4.5)	0.176	0.05788	o (o)
LiG	55 (56)	60	52	-0.3 (1.9)	-0.0 (1.6)	0.175	0.05999	o (o)
PrG	55 (55)	55	55	-1.4 (4.1)	-0.8 (2.9)	0.167	0.07119	o (o)
MCgG	54 (54)	55	54	-0.5 (2.6)	-0.1 (2.5)	0.158	0.08770	o (o)
PIns	55 (54)	50	58	-1.4 (3.4)	-1.0 (2.5)	0.148	0.11160	o (o)
Pallidum	58 (52)	21	83	0.3 (2.9)	-0.1 (2.2)	0.141	0.12864	o (o)
FRP	58 (57)	51	62	-3.9 (15.3)	-2.3 (14.0)	0.111	0.23076	o (o)
AccumbensArea	56 (49)	13	85	3.0 (50.3)	-0.5 (7.0)	0.109	0.23774	o (o)
VentralDC	53 (53)	54	53	-0.7 (2.0)	-0.5 (1.6)	0.107	0.24813	o (o)
GRe	56 (56)	55	57	-2.1 (6.7)	-1.5 (7.0)	0.097	0.29359	o (o)
CerebellarVermalLobulesVIII-X	54 (53)	52	55	-0.6 (2.8)	-0.4 (2.0)	0.096	0.30009	o (o)
PO	52 (52)	53	51	-0.1 (3.9)	-0.4 (2.7)	0.089	0.33509	o (o)
Cun	50 (51)	53	48	-0.3 (3.5)	-0.6 (3.9)	0.087	0.34872	o (o)
AOrG	53 (52)	49	55	-1.5 (5.1)	-1.1 (3.8)	0.086	0.35288	o (o)
CerebellarVermalLobulesI-V	49 (49)	47	51	-0.5 (2.5)	-0.3 (1.8)	0.083	0.37161	o (o)
OCP	53 (51)	46	57	-3.1 (10.4)	-2.2 (11.5)	0.081	0.38148	o (o)
Putamen	54 (49)	28	71	-0.1 (1.4)	-0.0 (0.8)	0.075	0.41924	o (o)
MPoG	50 (50)	50	50	-0.9 (8.4)	-0.5 (5.0)	0.057	0.54043	o (o)
BasalForebrain	51 (50)	47	53	-2.1 (12.9)	-1.6 (9.1)	0.046	0.62158	o (o)
PoG	50 (50)	52	49	-1.1 (4.8)	-0.9 (3.3)	0.045	0.62950	o (o)
CerebellumWhiteMatter	53 (50)	31	68	0.6 (7.2)	0.4 (5.3)	0.036	0.69917	o (o)
CerebellarVermalLobulesVI-VII	49 (48)	47	50	0.2 (4.3)	0.1 (3.7)	0.029	0.75272	o (o)
CO	48 (48)	47	48	-0.5 (3.7)	-0.6 (2.6)	0.025	0.78620	o (o)
Calc	48 (47)	45	50	-0.1 (3.5)	-0.2 (3.1)	0.019	0.83368	o (o)
STG	48 (48)	46	50	-0.6 (2.6)	-0.6 (1.8)	0.010	0.91377	o (o)

Longitudinal analysis (baseline→m12, pMCI vs. sMCI)

Table 7. Classification results in % (6-fold CV, LDA 100 runs, RF/SVM 20 runs) for distinguishing between pMCI and sMCI. Significant group differences indicated by + ($p < 0.05$) and ++ ($p < 0.001$). Bonferroni-corrected significance in parentheses. Sorted by effect size.

**progressive MCI** (N = 168, Positives^{P}) vs. **stable MCI** (N = 149, Negatives^{N}) **(longitudinal analysis, bl→m12)**								
structure	ACC (bACC)	SENS	SPEC	mean (SD) [%]^{P}	mean (SD) [%]^{N}	effect size (d)	p-value	sig. (corr.)
RandomForest (all features)	74 (73)	77	70					
SVM (all features)	74 (74)	72	75					
Age	49 (49)	46	51	73.6 (7.6)	74.0 (7.4)	0.050	0.65912	o (o)
Gender (female = 0, male = 1)	45 (45)	45	45	0.6 (0.5)	0.6 (0.5)	0.001	0.99199	o (o)
(surrogate structures)								
Ventricles	68 (69)	63	74	6.8 (4.3)	3.4 (3.1)	0.890	<0.00001	++ (++)
BrainTissue	63 (63)	64	63	-1.2 (1.0)	-0.6 (0.8)	0.652	<0.00001	++ (++)
CorticalGreyMatter	64 (64)	67	61	-1.8 (1.7)	-0.8 (1.3)	0.608	<0.00001	++ (++)
Brain	58 (58)	62	54	-0.8 (0.8)	-0.4 (0.6)	0.495	0.00002	++ (+)
DeepGreyMatter	64 (64)	62	66	-1.3 (1.2)	-0.7 (1.3)	0.428	0.00017	++ (+)
WhiteMatter	63 (63)	61	64	-0.6 (0.9)	-0.4 (0.7)	0.310	0.00615	+ (o)
(individual structures)								
Hippocampus	67 (67)	59	76	-4.1 (3.2)	-1.7 (2.2)	0.867	<0.00001	++ (++)
LateralVentricle	67 (68)	61	75	7.1 (4.6)	3.6 (3.4)	0.866	<0.00001	++ (++)
InfLatVent	69 (69)	63	75	6.7 (5.6)	2.6 (3.8)	0.845	<0.00001	++ (++)
LeftHippocampus	67 (68)	60	75	-4.2 (3.3)	-1.7 (2.5)	0.845	<0.00001	++ (++)
LeftLateralVentricle	66 (67)	60	73	7.2 (4.8)	3.6 (3.5)	0.836	<0.00001	++ (++)
RightLateralVentricle	67 (67)	62	72	7.0 (4.6)	3.6 (3.6)	0.828	<0.00001	++ (++)
MTG	70 (70)	65	75	-3.1 (2.4)	-1.4 (1.8)	0.807	<0.00001	++ (++)
ITG	67 (67)	64	70	-2.5 (2.0)	-1.1 (1.6)	0.793	<0.00001	++ (++)
LeftInfLatVent	68 (69)	62	76	6.7 (6.2)	2.4 (4.3)	0.783	<0.00001	++ (++)
EntA	67 (67)	67	68	-4.0 (3.7)	-1.4 (3.3)	0.749	<0.00001	++ (++)
PT	68 (68)	63	73	-5.5 (4.5)	-2.6 (3.3)	0.727	<0.00001	++ (++)
RightInfLatVent	64 (64)	58	71	6.7 (6.2)	2.7 (4.4)	0.726	<0.00001	++ (++)
FuG	66 (66)	63	69	-1.5 (1.6)	-0.5 (1.1)	0.694	<0.00001	++ (++)
PP	66 (67)	64	70	-5.8 (4.6)	-2.9 (3.9)	0.692	<0.00001	++ (++)
RightHippocampus	65 (65)	57	73	-4.0 (3.9)	-1.7 (2.8)	0.677	<0.00001	++ (++)
3rdVentricle	64 (64)	61	67	4.7 (3.8)	2.5 (3.7)	0.590	<0.00001	++ (++)
Amygdala	65 (66)	58	73	-3.2 (4.1)	-1.2 (2.8)	0.577	<0.00001	++ (++)
RightAmygdala	64 (64)	56	73	-3.7 (5.5)	-1.1 (3.3)	0.567	<0.00001	++ (++)
TTG	61 (62)	61	62	-4.4 (4.2)	-2.2 (3.5)	0.558	<0.00001	++ (++)
TMP	64 (64)	64	64	-3.9 (3.7)	-2.2 (3.0)	0.524	<0.00001	++ (++)
Caudate	62 (62)	62	62	-4.8 (5.2)	-2.4 (3.9)	0.510	<0.00001	++ (++)
SPL	64 (64)	67	62	-2.1 (2.8)	-0.7 (2.9)	0.502	0.00001	++ (++)
SMG	62 (62)	61	62	-2.6 (2.9)	-1.3 (2.5)	0.489	0.00002	++ (+)
PHG	58 (58)	57	60	-1.7 (2.2)	-0.7 (1.9)	0.478	0.00003	++ (+)
MOG	58 (58)	51	65	-2.0 (3.2)	-0.8 (2.1)	0.442	0.00011	++ (+)
AnG	61 (61)	59	63	-2.1 (2.9)	-1.0 (2.2)	0.432	0.00015	++ (+)
MSFG	59 (59)	58	59	-2.2 (3.0)	-1.0 (2.4)	0.428	0.00017	++ (+)
AIns	56 (56)	55	57	-1.5 (2.2)	-0.7 (1.8)	0.387	0.00065	++ (o)
LeftAmygdala	62 (63)	53	72	-2.8 (4.5)	-1.3 (3.5)	0.376	0.00095	++ (o)
TrIFG	60 (60)	57	63	-2.6 (3.0)	-1.6 (2.5)	0.366	0.00127	+ (o)
BrainStem	62 (61)	68	55	-0.8 (0.9)	-0.3 (1.7)	0.362	0.00144	+ (o)
IOG	56 (57)	52	62	-1.8 (2.5)	-1.0 (2.1)	0.347	0.00220	+ (o)
SMC	54 (54)	53	55	-1.7 (2.9)	-0.8 (2.4)	0.333	0.00328	+ (o)
OCP	57 (57)	53	61	-4.4 (13.1)	-0.5 (10.0)	0.332	0.00337	+ (o)
OrIFG	55 (55)	51	59	-3.0 (3.4)	-1.9 (2.9)	0.332	0.00339	+ (o)
SFG	58 (58)	55	61	-2.1 (3.0)	-1.1 (2.9)	0.318	0.00498	+ (o)
MFG	58 (58)	58	58	-1.9 (2.7)	-1.1 (2.4)	0.317	0.00516	+ (o)
PCgG	58 (58)	52	63	-0.7 (1.8)	-0.1 (1.6)	0.314	0.00562	+ (o)
PCu	55 (56)	53	58	-1.0 (2.5)	-0.4 (1.9)	0.295	0.00923	+ (o)
CerebralWhiteMatter	62 (63)	60	65	-0.6 (0.8)	-0.4 (0.7)	0.284	0.01201	+ (o)
FO	55 (55)	51	60	-1.9 (3.3)	-1.0 (2.8)	0.284	0.01224	+ (o)
ThalamusProper	65 (65)	55	76	-2.6 (7.3)	-1.1 (2.8)	0.273	0.01582	+ (o)
LiG	55 (55)	54	56	-0.4 (1.5)	-0.0 (1.4)	0.268	0.01794	+ (o)
OFuG	54 (54)	51	57	-1.2 (2.9)	-0.5 (2.8)	0.249	0.02748	+ (o)
MOrG	55 (55)	53	56	-1.6 (2.6)	-0.9 (3.1)	0.245	0.02999	+ (o)
LOrG	54 (54)	53	55	-2.4 (4.1)	-1.3 (4.7)	0.245	0.03029	+ (o)
Cun	53 (53)	53	54	-0.5 (3.6)	0.3 (3.0)	0.231	0.04104	+ (o)
SOG	55 (55)	55	55	-1.6 (4.7)	-0.6 (3.9)	0.229	0.04284	+ (o)
SCA	57 (57)	58	56	-2.9 (6.8)	-1.4 (7.1)	0.225	0.04654	+ (o)
MFC	53 (53)	51	56	-1.4 (3.9)	-0.5 (4.1)	0.222	0.04980	+ (o)
OpIFG	58 (58)	60	56	-1.7 (3.2)	-1.1 (2.3)	0.217	0.05489	o (o)
GRe	53 (53)	56	51	-1.9 (5.4)	-0.5 (7.2)	0.215	0.05712	o (o)
AOrG	52 (53)	47	58	-2.1 (4.1)	-1.2 (4.0)	0.210	0.06272	o (o)
POrG	55 (55)	55	54	-0.8 (2.9)	-0.2 (2.7)	0.210	0.06305	o (o)
CSF	53 (53)	52	55	1.0 (3.4)	0.4 (3.6)	0.186	0.10005	o (o)
PrG	55 (55)	59	51	-1.1 (2.9)	-0.6 (3.1)	0.173	0.12492	o (o)
Putamen	48 (50)	18	82	-0.4 (3.5)	0.1 (1.2)	0.170	0.13126	o (o)
PIns	54 (54)	53	55	-0.9 (2.8)	-0.5 (1.9)	0.168	0.13708	o (o)
AccumbensArea	55 (56)	38	75	-1.3 (9.7)	0.1 (7.8)	0.167	0.13805	o (o)
ACg	52 (53)	47	58	-1.3 (2.7)	-0.9 (2.7)	0.160	0.15511	o (o)
CerebellarVermalLobulesI-V	55 (55)	51	59	-0.6 (1.3)	-0.3 (2.1)	0.153	0.17461	o (o)
CO	52 (52)	48	56	-0.7 (2.9)	-0.3 (2.3)	0.150	0.18303	o (o)
BasalForebrain	54 (54)	55	53	-1.5 (10.3)	-0.1 (9.8)	0.138	0.22106	o (o)
CerebellumExterior	55 (56)	52	59	-1.0 (1.6)	-0.8 (2.1)	0.137	0.22484	o (o)
CerebellumWhiteMatter	56 (55)	66	44	-0.3 (3.3)	0.2 (4.1)	0.128	0.25500	o (o)
STG	52 (52)	57	48	-0.6 (2.4)	-0.3 (1.8)	0.127	0.26171	o (o)
Calc	52 (52)	54	51	-0.3 (3.4)	0.0 (2.4)	0.126	0.26340	o (o)
MCgG	49 (48)	51	46	-0.3 (2.2)	-0.0 (2.2)	0.118	0.29487	o (o)
PoG	52 (52)	55	49	-1.1 (3.9)	-0.8 (2.9)	0.095	0.39793	o (o)
MPoG	50 (50)	54	45	-0.1 (7.2)	0.4 (5.0)	0.091	0.42142	o (o)
FRP	48 (48)	54	41	-2.4 (9.7)	-1.6 (16.5)	0.056	0.62168	o (o)
CerebellarVermalLobulesVIII-X	48 (48)	47	49	-0.4 (2.4)	-0.5 (2.8)	0.056	0.62187	o (o)
PO	49 (49)	51	48	-0.1 (3.7)	0.0 (2.8)	0.052	0.64691	o (o)
Pallidum	49 (50)	38	62	-0.1 (3.0)	0.0 (3.0)	0.051	0.65324	o (o)
CerebellarVermalLobulesVI-VII	47 (47)	46	47	-0.1 (4.1)	0.1 (3.7)	0.050	0.65863	o (o)
MPrG	47 (47)	47	46	-0.5 (2.8)	-0.5 (2.8)	0.028	0.80310	o (o)
VentralDC	49 (49)	51	47	-0.6 (1.9)	-0.5 (3.1)	0.021	0.85372	o (o)
4thVentricle	46 (47)	45	48	2.1 (7.0)	2.0 (5.5)	0.018	0.87648	o (o)

Longitudinal analysis (baseline→m24, AD vs. HC)

Table 8. Classification results in % (6-fold CV, LDA 100 runs, RF/SVM 20 runs) for distinguishing between AD and HC. Significant group differences indicated by + ($p < 0.05$) and ++ ($p < 0.001$). Bonferroni-corrected significance in parentheses. Sorted by effect size.

**AD patients** (N=117, Positives^P) vs. **Healthy Controls** (N=168, Negatives^N) **(longitudinal analysis, bl→m24)**								
structure	ACC (bACC)	SENS	SPEC	mean (SD) [%]^P	mean (SD) [%]^N	effect size (d)	p-value	sig. (corr.)
RandomForest (all features)	89 (88)	81	94					
SVM (all features)	89 (88)	80	95					
Age	47 (47)	46	47	75.5 (7.7)	75.9 (5.2)	0.061	0.61542	o (o)
Gender (female=0, male=1)	49 (48)	44	52	0.5 (0.5)	0.6 (0.5)	0.059	0.62275	o (o)
(surrogate structures)								
Ventricles	83 (81)	70	92	14.7 (7.9)	5.8 (3.7)	1.531	<0.00001	++ (++)
BrainTissue	80 (78)	71	86	-2.6 (1.3)	-1.1 (0.8)	1.353	<0.00001	++ (++)
WhiteMatter	75 (74)	65	82	-1.8 (1.4)	-0.6 (0.8)	1.121	<0.00001	++ (++)
CorticalGreyMatter	73 (73)	69	77	-3.5 (2.4)	-1.6 (1.7)	0.928	<0.00001	++ (++)
Brain	68 (67)	65	70	-1.5 (1.0)	-0.8 (0.7)	0.847	<0.00001	++ (++)
DeepGreyMatter	67 (66)	63	69	-2.3 (1.8)	-1.3 (1.3)	0.604	<0.00001	++ (++)
(individual structures)								
Hippocampus	87 (85)	77	94	-10.2 (6.2)	-2.0 (2.4)	1.880	<0.00001	++ (++)
InfLatVent	84 (83)	76	90	15.9 (8.8)	3.5 (4.5)	1.871	<0.00001	++ (++)
RightInfLatVent	83 (82)	73	90	16.1 (9.7)	3.5 (4.9)	1.728	<0.00001	++ (++)
LeftHippocampus	86 (84)	77	92	-9.9 (6.6)	-1.9 (2.7)	1.701	<0.00001	++ (++)
LeftInfLatVent	80 (78)	69	88	15.5 (9.5)	3.5 (5.2)	1.652	<0.00001	++ (++)
MTG	83 (81)	70	92	-6.8 (4.1)	-2.0 (1.6)	1.651	<0.00001	++ (++)
RightHippocampus	84 (81)	69	94	-10.5 (7.6)	-2.0 (2.6)	1.617	<0.00001	++ (++)
ITG	83 (81)	71	91	-5.3 (3.3)	-1.6 (1.4)	1.603	<0.00001	++ (++)
FuG	85 (83)	72	93	-3.7 (2.7)	-0.7 (0.9)	1.588	<0.00001	++ (++)
LeftLateralVentricle	82 (80)	71	89	15.6 (8.2)	6.5 (4.4)	1.470	<0.00001	++ (++)
PP	80 (79)	73	85	-10.9 (6.1)	-3.9 (3.5)	1.466	<0.00001	++ (++)
LateralVentricle	80 (78)	65	90	15.4 (8.5)	6.3 (4.0)	1.454	<0.00001	++ (++)
TMP	84 (82)	75	90	-8.2 (5.1)	-2.8 (2.5)	1.430	<0.00001	++ (++)
RightLateralVentricle	80 (77)	63	92	15.1 (9.3)	6.2 (4.0)	1.331	<0.00001	++ (++)
PT	78 (77)	70	83	-10.0 (6.2)	-3.8 (3.5)	1.276	<0.00001	++ (++)
EntA	80 (79)	72	86	-7.0 (5.5)	-2.2 (3.1)	1.151	<0.00001	++ (++)
CerebralWhiteMatter	75 (74)	65	83	-1.9 (1.3)	-0.7 (0.9)	1.138	<0.00001	++ (++)
Amygdala	81 (79)	70	89	-7.3 (6.7)	-1.7 (3.7)	1.076	<0.00001	++ (++)
RightAmygdala	79 (77)	69	85	-7.6 (7.0)	-1.8 (4.2)	1.050	<0.00001	++ (++)
SMG	71 (70)	61	78	-5.7 (4.6)	-2.2 (2.6)	0.968	<0.00001	++ (++)
AIns	73 (73)	67	78	-3.6 (3.4)	-1.1 (2.0)	0.965	<0.00001	++ (++)
3rdVentricle	70 (69)	65	74	8.4 (5.7)	4.1 (4.0)	0.904	<0.00001	++ (++)
PHG	73 (71)	65	78	-3.4 (3.7)	-0.9 (2.0)	0.901	<0.00001	++ (++)
LeftAmygdala	79 (77)	65	89	-7.1 (8.7)	-1.7 (3.9)	0.863	<0.00001	++ (++)
ThalamusProper	78 (77)	67	86	-4.5 (3.5)	-2.0 (2.8)	0.838	<0.00001	++ (++)
MSFG	71 (70)	68	73	-4.7 (3.9)	-2.1 (2.8)	0.789	<0.00001	++ (++)
PCgG	67 (65)	57	73	-2.1 (2.7)	-0.6 (1.7)	0.729	<0.00001	++ (++)
AnG	69 (67)	60	75	-4.5 (4.4)	-2.0 (2.7)	0.718	<0.00001	++ (++)
FO	69 (68)	58	77	-4.8 (5.5)	-1.8 (3.0)	0.706	<0.00001	++ (++)
TTG	70 (70)	65	74	-7.0 (6.8)	-3.4 (3.3)	0.705	<0.00001	++ (++)
IOG	64 (63)	57	70	-3.8 (3.5)	-1.8 (2.5)	0.671	<0.00001	++ (++)
POrG	61 (61)	63	59	-2.7 (3.0)	-0.9 (2.7)	0.628	<0.00001	++ (++)
PCu	69 (67)	58	76	-2.7 (3.7)	-1.0 (2.2)	0.601	<0.00001	++ (++)
SMC	62 (62)	57	66	-3.9 (3.9)	-2.0 (2.5)	0.595	<0.00001	++ (++)
OrIFG	63 (62)	58	67	-4.8 (4.7)	-2.5 (3.6)	0.573	<0.00001	++ (++)
MFG	61 (60)	58	63	-3.5 (3.8)	-1.9 (2.6)	0.521	0.00002	++ (+)
MOrG	64 (63)	61	66	-3.3 (4.5)	-1.4 (3.1)	0.509	0.00003	++ (+)
ACg	62 (60)	50	70	-3.3 (4.0)	-1.7 (2.4)	0.505	0.00004	++ (+)
BrainStem	60 (59)	53	65	-1.5 (1.4)	-1.0 (1.0)	0.456	0.00018	++ (+)
MOG	61 (60)	58	63	-3.1 (4.0)	-1.3 (4.2)	0.452	0.00021	++ (+)
SPL	64 (64)	63	65	-3.6 (5.0)	-1.7 (3.9)	0.446	0.00025	++ (+)
TrIFG	62 (60)	54	67	-3.8 (3.5)	-2.4 (2.9)	0.443	0.00028	++ (+)
LOrG	62 (61)	53	69	-4.9 (6.1)	-2.5 (5.5)	0.426	0.00047	++ (+)
OpIFG	60 (59)	57	62	-3.5 (3.7)	-2.1 (2.9)	0.423	0.00051	++ (+)
CSF	59 (58)	58	59	2.0 (5.1)	0.1 (4.1)	0.412	0.00072	++ (o)
SCA	63 (63)	61	64	-5.9 (13.2)	-2.1 (6.2)	0.388	0.00141	+ (o)
MFC	61 (60)	55	65	-3.4 (5.6)	-1.7 (4.0)	0.354	0.00356	+ (o)
GRe	64 (63)	61	66	-4.3 (8.0)	-1.7 (6.9)	0.347	0.00427	+ (o)
OFuG	59 (59)	60	58	-1.6 (3.1)	-0.6 (3.0)	0.331	0.00636	+ (o)
Caudate	61 (61)	58	63	-5.9 (9.5)	-3.6 (4.4)	0.328	0.00683	+ (o)
STG	55 (55)	52	58	-1.7 (3.2)	-0.9 (2.0)	0.306	0.01145	+ (o)
PrG	58 (58)	60	57	-2.7 (3.8)	-1.8 (3.4)	0.252	0.03747	+ (o)
AOrG	54 (54)	53	55	-2.6 (4.9)	-1.6 (3.4)	0.243	0.04413	+ (o)
4thVentricle	60 (59)	55	63	3.8 (7.0)	2.3 (5.8)	0.240	0.04731	+ (o)
SFG	57 (57)	59	55	-2.7 (4.0)	-2.0 (3.2)	0.209	0.08426	o (o)
CerebellumExterior	60 (60)	56	63	-1.9 (2.9)	-1.4 (2.0)	0.200	0.09756	o (o)
LiG	53 (53)	56	51	-0.8 (3.1)	-0.3 (2.3)	0.193	0.10963	o (o)
Putamen	59 (53)	22	84	-0.4 (3.0)	0.1 (2.0)	0.192	0.11187	o (o)
MCgG	57 (57)	56	57	-1.3 (3.4)	-0.7 (2.3)	0.190	0.11579	o (o)
VentralDC	57 (57)	53	61	-1.3 (1.8)	-1.0 (2.4)	0.180	0.13700	o (o)
SOG	58 (58)	59	56	-2.6 (5.8)	-1.5 (6.6)	0.175	0.14836	o (o)
Pallidum	52 (53)	62	45	0.2 (3.5)	-0.2 (2.0)	0.164	0.17299	o (o)
CerebellarVermalLobulesI-V	54 (52)	43	62	-1.3 (3.5)	-0.7 (4.4)	0.164	0.17424	o (o)
PIns	54 (53)	52	55	-1.7 (4.1)	-1.2 (2.7)	0.141	0.24371	o (o)
PO	52 (51)	48	54	-1.6 (4.6)	-1.2 (3.2)	0.109	0.36791	o (o)
FRP	58 (57)	50	63	-5.3 (18.9)	-3.6 (14.8)	0.107	0.37505	o (o)
CO	53 (52)	48	56	-1.9 (4.2)	-1.6 (3.1)	0.105	0.38495	o (o)
Cun	54 (53)	49	58	-0.1 (4.8)	-0.6 (7.4)	0.077	0.52528	o (o)
MPoG	48 (48)	44	51	-1.1 (8.5)	-1.6 (5.5)	0.074	0.54109	o (o)
BasalForebrain	48 (48)	48	48	-2.6 (16.4)	-3.4 (9.7)	0.062	0.60717	o (o)
AccumbensArea	58 (52)	18	86	2.6 (60.0)	0.1 (16.1)	0.061	0.61555	o (o)
CerebellumWhiteMatter	49 (52)	69	35	0.1 (7.1)	0.4 (4.8)	0.057	0.63523	o (o)
MPrG	48 (48)	46	50	-1.8 (3.9)	-1.7 (3.7)	0.043	0.71987	o (o)
CerebellarVermalLobulesVIII-X	46 (47)	53	40	-1.0 (3.0)	-1.2 (6.2)	0.039	0.74661	o (o)
PoG	48 (47)	47	48	-1.9 (5.0)	-1.7 (4.1)	0.027	0.82435	o (o)
OCP	49 (49)	48	49	-2.4 (17.0)	-2.1 (17.1)	0.015	0.90207	o (o)
CerebellarVermalLobulesVI-VII	47 (47)	46	48	-0.3 (4.7)	-0.4 (4.5)	0.014	0.90530	o (o)
Calc	47 (46)	43	49	-0.8 (4.8)	-0.8 (3.3)	0.013	0.91102	o (o)

Longitudinal analysis (baseline→m24, pMCI vs. sMCI)

Table 9. Classification results in % (6-fold CV, LDA 100 runs, RF/SVM 20 runs) for distinguishing between pMCI and sMCI. Significant group differences indicated by + ($p < 0.05$) and ++ ($p < 0.001$). Bonferroni-corrected significance in parentheses. Sorted by effect size.

**progressive MCI** (N = 140, Positives^P) vs. **stable MCI** (N = 107, Negatives^N) **(longitudinal analysis, bl→m24)**								
structure	ACC (bACC)	SENS	SPEC	mean (SD) [%]^P	mean (SD) [%]^N	effect size (d)	p-value	sig. (corr.)
RandomForest (all features)	79 (78)	82	74					
SVM (all features)	76 (76)	76	76					
Age	54 (54)	51	58	73.3 (7.8)	74.7 (7.5)	0.176	0.17108	o (o)
Gender (female = 0, male = 1)	46 (47)	40	54	0.6 (0.5)	0.6 (0.5)	0.053	0.67739	o (o)
(surrogate structures)								
Ventricles	71 (72)	65	80	13.7 (7.4)	7.1 (5.6)	0.989	<0.00001	++ (++)
BrainTissue	68 (69)	62	76	-2.3 (1.5)	-1.2 (1.0)	0.845	<0.00001	++ (++)
DeepGreyMatter	66 (67)	64	69	-2.1 (1.4)	-1.2 (1.0)	0.714	<0.00001	++ (++)
CorticalGreyMatter	65 (66)	61	71	-3.2 (2.5)	-1.7 (1.6)	0.707	<0.00001	++ (++)
Brain	63 (63)	60	67	-1.5 (1.1)	-0.8 (0.7)	0.707	<0.00001	++ (++)
WhiteMatter	64 (65)	55	74	-1.6 (1.3)	-0.8 (1.0)	0.661	<0.00001	++ (++)
(individual structures)								
FuG	75 (76)	69	83	-3.1 (2.1)	-1.0 (1.4)	1.141	<0.00001	++ (++)
Hippocampus	73 (74)	68	80	-8.9 (5.1)	-3.7 (3.8)	1.118	<0.00001	++ (++)
LeftHippocampus	74 (74)	72	77	-9.3 (5.6)	-3.8 (4.2)	1.083	<0.00001	++ (++)
EntA	71 (72)	66	77	-7.4 (4.7)	-2.8 (3.6)	1.070	<0.00001	++ (++)
InfLatVent	70 (72)	62	81	14.6 (8.9)	6.2 (6.6)	1.053	<0.00001	++ (++)
MTG	72 (74)	64	83	-6.1 (3.9)	-2.6 (2.6)	1.016	<0.00001	++ (++)
LeftInfLatVent	68 (69)	60	79	14.7 (9.4)	6.4 (6.8)	0.987	<0.00001	++ (++)
PT	68 (68)	62	75	-9.7 (5.6)	-4.7 (4.2)	0.971	<0.00001	++ (++)
LateralVentricle	70 (71)	64	78	14.4 (7.9)	7.5 (6.0)	0.960	<0.00001	++ (++)
ITG	72 (73)	66	80	-4.7 (2.9)	-2.1 (2.2)	0.958	<0.00001	++ (++)
LeftLateralVentricle	69 (70)	62	77	14.6 (8.2)	7.6 (6.1)	0.956	<0.00001	++ (++)
RightInfLatVent	66 (68)	60	76	14.3 (10.1)	5.9 (7.4)	0.923	<0.00001	++ (++)
RightHippocampus	71 (72)	65	79	-8.6 (5.7)	-3.7 (4.6)	0.920	<0.00001	++ (++)
RightLateralVentricle	69 (70)	61	79	14.1 (8.1)	7.5 (6.3)	0.907	<0.00001	++ (++)
Amygdala	67 (68)	61	76	-6.4 (5.2)	-2.9 (3.5)	0.770	<0.00001	++ (++)
TTG	65 (66)	62	70	-8.0 (5.3)	-4.4 (4.1)	0.752	<0.00001	++ (++)
PHG	66 (68)	58	77	-3.5 (2.8)	-1.5 (2.4)	0.746	<0.00001	++ (++)
3rdVentricle	64 (65)	61	68	9.1 (6.2)	4.7 (5.5)	0.740	<0.00001	++ (++)
PP	68 (69)	65	72	-10.0 (5.7)	-5.9 (5.5)	0.728	<0.00001	++ (++)
TMP	68 (69)	64	74	-6.9 (4.5)	-3.9 (3.6)	0.721	<0.00001	++ (++)
RightAmygdala	67 (68)	62	73	-6.5 (6.3)	-2.7 (4.3)	0.688	<0.00001	++ (++)
CerebralWhiteMatter	65 (66)	58	73	-1.6 (1.3)	-0.9 (1.0)	0.635	<0.00001	++ (++)
SMG	62 (64)	54	74	-4.8 (4.0)	-2.5 (2.8)	0.633	<0.00001	++ (++)
LeftAmygdala	65 (66)	60	72	-6.4 (6.0)	-3.2 (4.2)	0.605	<0.00001	++ (++)
PCgG	62 (62)	57	67	-1.7 (2.3)	-0.5 (1.6)	0.577	0.00001	++ (++)
MOG	63 (64)	55	74	-3.3 (4.1)	-1.3 (2.8)	0.570	0.00001	++ (+)
MSFG	63 (63)	59	67	-4.2 (4.0)	-2.2 (3.0)	0.551	0.00003	++ (+)
AIns	58 (59)	53	66	-2.8 (3.0)	-1.4 (2.0)	0.529	0.00005	++ (+)
SCA	65 (65)	69	61	-5.8 (8.0)	-2.1 (7.1)	0.480	0.00023	++ (+)
AnG	61 (62)	56	69	-3.9 (4.9)	-2.0 (2.8)	0.478	0.00024	++ (+)
BrainStem	58 (58)	59	57	-1.3 (1.4)	-0.6 (1.4)	0.462	0.00038	++ (+)
FO	61 (63)	52	73	-4.5 (5.6)	-2.3 (3.7)	0.462	0.00039	++ (+)
CerebellumExterior	65 (65)	64	66	-1.9 (2.0)	-1.1 (1.3)	0.434	0.00085	++ (o)
MFG	58 (59)	54	64	-3.3 (3.7)	-2.0 (2.4)	0.432	0.00090	++ (o)
PCu	61 (62)	58	65	-2.3 (3.9)	-1.0 (2.0)	0.430	0.00093	++ (o)
IOG	63 (64)	59	69	-3.1 (3.8)	-1.7 (2.4)	0.417	0.00133	+ (o)
SPL	62 (61)	63	59	-3.4 (4.7)	-1.7 (3.3)	0.408	0.00167	+ (o)
LiG	58 (57)	59	55	-0.9 (2.2)	-0.1 (2.0)	0.405	0.00181	+ (o)
MOrG	59 (59)	58	60	-3.0 (2.9)	-1.8 (3.5)	0.398	0.00218	+ (o)
OCP	59 (58)	61	56	-3.9 (11.9)	0.0 (9.7)	0.352	0.00660	+ (o)
AccumbensArea	63 (65)	52	77	-2.3 (5.5)	-0.5 (5.1)	0.342	0.00827	+ (o)
ThalamusProper	66 (67)	56	79	-4.7 (8.7)	-2.4 (2.5)	0.341	0.00844	+ (o)
SMC	54 (55)	51	59	-3.3 (3.8)	-2.1 (2.8)	0.335	0.00972	+ (o)
TrIFG	58 (59)	52	65	-3.7 (3.7)	-2.7 (2.6)	0.320	0.01334	+ (o)
CSF	58 (58)	60	56	1.3 (5.1)	-0.1 (4.0)	0.318	0.01404	+ (o)
LOrG	57 (58)	53	62	-4.2 (4.4)	-2.8 (5.1)	0.309	0.01673	+ (o)
OrIFG	59 (59)	53	65	-4.5 (5.0)	-3.1 (3.4)	0.309	0.01695	+ (o)
Caudate	59 (59)	57	61	-5.6 (8.1)	-3.5 (5.8)	0.302	0.01965	+ (o)
CerebellumWhiteMatter	61 (59)	73	45	-0.6 (2.7)	0.3 (3.3)	0.300	0.02030	+ (o)
STG	58 (58)	52	65	-1.5 (3.3)	-0.7 (2.1)	0.294	0.02284	+ (o)
OpIFG	58 (58)	53	63	-3.1 (4.5)	-2.0 (2.4)	0.287	0.02644	+ (o)
AOrG	55 (56)	51	60	-3.2 (5.1)	-1.9 (4.0)	0.285	0.02752	+ (o)
SOG	57 (57)	56	57	-2.9 (5.7)	-1.6 (3.7)	0.269	0.03737	+ (o)
ACg	55 (55)	52	59	-2.7 (3.8)	-1.8 (3.1)	0.261	0.04334	+ (o)
SFG	59 (60)	56	64	-2.9 (3.8)	-2.0 (2.8)	0.253	0.05003	o (o)
Calc	58 (57)	64	51	-0.7 (4.9)	0.4 (4.4)	0.241	0.06227	o (o)
MFC	58 (60)	49	70	-3.9 (5.9)	-2.6 (4.6)	0.237	0.06573	o (o)
POrG	55 (55)	55	55	-2.0 (3.2)	-1.3 (2.8)	0.236	0.06716	o (o)
GRe	59 (60)	52	67	-4.4 (6.6)	-3.0 (5.7)	0.229	0.07521	o (o)
PrG	55 (55)	56	53	-2.2 (3.7)	-1.4 (3.0)	0.229	0.07632	o (o)
CO	54 (54)	50	59	-1.8 (4.5)	-1.0 (3.0)	0.193	0.13491	o (o)
PIns	53 (54)	51	57	-1.8 (3.2)	-1.3 (2.4)	0.192	0.13540	o (o)
OFuG	57 (57)	57	58	-1.5 (3.7)	-1.0 (2.3)	0.166	0.19645	o (o)
PO	56 (56)	53	59	-1.2 (5.0)	-0.5 (3.5)	0.165	0.19971	o (o)
FRP	56 (56)	55	57	-4.9 (13.3)	-3.0 (16.1)	0.129	0.31612	o (o)
CerebellarVermalLobulesI-V	56 (56)	54	58	-1.1 (2.1)	-0.8 (2.1)	0.128	0.31896	o (o)
Pallidum	48 (52)	23	81	0.4 (4.3)	-0.0 (2.1)	0.118	0.35737	o (o)
CerebellarVermalLobulesVIII-X	51 (52)	49	55	-1.1 (2.7)	-0.8 (2.2)	0.118	0.36012	o (o)
MCgG	51 (51)	51	50	-1.1 (2.9)	-0.8 (2.4)	0.117	0.36161	o (o)
PoG	52 (52)	52	52	-1.7 (4.7)	-1.4 (3.2)	0.092	0.47252	o (o)
CerebellarVermalLobulesVI-VII	51 (51)	49	52	-0.8 (4.7)	-0.4 (4.2)	0.085	0.50816	o (o)
4thVentricle	54 (54)	50	58	2.7 (7.4)	2.2 (6.0)	0.076	0.55268	o (o)
MPrG	51 (51)	54	48	-1.5 (3.6)	-1.3 (3.0)	0.072	0.57329	o (o)
BasalForebrain	47 (46)	48	45	-2.7 (13.1)	-1.9 (10.0)	0.066	0.60690	o (o)
VentralDC	51 (51)	52	50	-1.1 (2.5)	-0.9 (2.3)	0.060	0.64346	o (o)
Putamen	47 (49)	40	57	-0.1 (2.0)	-0.0 (0.7)	0.034	0.79321	o (o)
Cun	46 (46)	45	47	-0.1 (4.3)	-0.1 (2.6)	0.003	0.98036	o (o)
MPoG	47 (47)	46	47	-0.8 (6.7)	-0.8 (4.1)	0.001	0.99373	o (o)
